# *GhGPAT12*/*25* Are Essential for the Formation of Anther Cuticle and Pollen Exine in Cotton (*Gossypium hirsutum* L.)

**DOI:** 10.3389/fpls.2021.667739

**Published:** 2021-05-13

**Authors:** Meng Zhang, Hengling Wei, Pengbo Hao, Aimin Wu, Qiang Ma, Jingjing Zhang, Hantao Wang, Xiaokang Fu, Liang Ma, Jianhua Lu, Shuxun Yu

**Affiliations:** ^1^State Key Laboratory of Cotton Biology, Institute of Cotton Research, Chinese Academy of Agricultural Sciences, Anyang, China; ^2^College of Biology and Food Engineering, Anyang Institute of Technology, Anyang, China

**Keywords:** *GhGPAT12/25*, cotton, CRISPR/Cas9, male sterility, anther cuticle, pollen exine

## Abstract

Glycerol-3-phosphate acyltransferases (GPATs), critical for multiple biological processes like male fertility, have been extensively characterized. However, their precise functions and underlying regulatory mechanism in cotton anther development are unclear. This research demonstrated the importance of *GhGPAT12*/*25* (a paralogs pair on A12/D12 sub-chromosome of cotton) to regulate the degradation of tapetum, anther cuticle formation, and pollen exine development. *GhGPAT12* and *GhGPAT25* exhibited specifically detected transcripts in tapetum and pollen exine during the early anther developmental stages. GhGPAT12/25 are sn-2 glycerol-3-phosphate acyltransferases and can transfer the acyl group of palmitoyl-CoA to glycerol-3-phosphate (G3P). CRISPR/Cas9-mediated knockout identified the functional redundancy of *GhGPAT12* and *GhGPAT25*. Knockout of both genes caused completely male sterility associated with abnormal anther cuticle, swollen tapetum, and inviable microspores with defective exine and irregular unrestricted shape. RNA-seq analysis showed that the loss of function of *GhGPAT12*/*25* affects the processes of wax metabolic, glycerol monomer biosynthesis, and transport. Consistently, cuticular waxes were dramatically reduced in mutant anthers. Yeast one-hybrid system (Y1H), virus-induced gene silencing (VIGS), and dual-luciferase (LUC) assays illustrated that GhMYB80s are likely to directly activate the expression of *GhGPAT12*/*25*. This study provides important insights for revealing the regulatory mechanism underlying anther development in cotton.

## Introduction

In flowering plants, male reproductive development is a complex biological process involving the differentiation of stamen primordium to produce anther, the transition from sporophytic to gametophytic generation, and the formation of pollen grains (Scott et al., 2004). The formed anther consists of four somatic layers (from the surface to the interior): the epidermis, endothecium, middle layer, and the tapetum (Goldberg et al., 1995). Microspore mother cells localized at the center of the anther lobes will differentiate and undergo meiosis to produce microspores, which then give rise to pollen grains. Pollen development is intimately linked to the morphogenesis of anther and requires the synergistic interaction between sporophytic and gametophytic genes (Goldberg et al., 1995; Scott et al., 2004).

To ensure the biological processes of microspore development and pollen grain maturation, plants establish two barriers in order to resist various environmental stresses. One of the barriers is the anther cuticle continuously coating the outermost surface of anther epidermis (Piffanelli et al., 1998). Similarly to the epidermal cuticle of vegetative organs, anther cuticle is a complex biopolymer composed of two types of lipophilic materials: cutin and waxes (Kolattukudy, 2001). The hydrophobic cutin matrix is synthesized by hydroxyl and epoxy fatty acids, and it determines the framework of the anther cuticle (Kolattukudy, 2001; Heredia, 2003). Waxes contain different substances generated from (very)-long-chain fatty acids through alcohol/alkane-forming pathways, and they are embedded or/and overloaded on the surface of cutic matrix (Kunst and Samuels, 2003). The synthetic pathway of the cuticle involves the synthesis, transport, and polymerization of cutin monomers. Genetic and biochemical evidences have demonstrate that C16 and C18 fatty acids are the main substrates for cutin formation, and they are firstly esterified to produce (fatty) acyl-CoA (Kunst and Samuels, 2003; Zhang et al., 2018). This formation process is catalyzed by long-chain acyl-CoA synthetases (LACS), like *LACS1* and *LACS2* in *Arabidopsis* (Lu et al., 2009). Then, the products are oxidized by CYP86 and CYP77 subfamilies members, such as the CYP703A2/CYP703A3 and CYP704B1/CYP704B2 in *Arabidopsis*/rice (Morant et al., 2007; Dobritsa et al., 2009; Li et al., 2010; Yang et al., 2014). Furthermore, the acyl group of acyl-CoA is transferred to glycerol-3-phosphate (G3P) under the catalysis of glycerol-3-phosphate acyltransferase (GPAT), thus yielding monoacylglycerol cutin monomers (Li-Beisson et al., 2013). *OsGPAT3* and *ZmMS33* were reported to be genes involved in this aspect for anther cutin formation (Men et al., 2017; Zhang et al., 2018). ATP-binding cassette (ABC) transporters (i.e., ABCG11 and ABCG12) are then recruited for the channeling of glycerol monomers through the plasma membrane (Pighin et al., 2004; Panikashvili et al., 2007). Subsequently, the hydrophobic polymer compounds will pass through the apoplastic compartment or cell wall; lipid transfer proteins (LTPs) collaborate in the completion of this process (Edstam and Edqvist, 2014). Pollen wall, especially the exine, is the second barrier practically for pollen grain maturation and it is also indispensable for pollen-stigma communication (Piffanelli et al., 1998). The main component of the exine is sporopollenin (an extremely chemically inert biopolymer), which was reported to be likely synthesized by polyvinyl alcohol units and 7-O-p-coumaroylated C16 aliphatic units in pine (Li et al., 2019a). The synthesis and secretion of the major lipidic precursors of cutin and sporopollenin largely rely on the developing tapetum. Mutations in genes responsible for the formation or transport of these lipid components usually cause abnormal programmed cell death (PCD) in the tapetum. Examples of such genes are *AMS*/*TDR*, *MYB80*, *MS1*/*PTC1*, *MS2*/*DPW*, *CYP703A2*/*CYP703A3*, and *CYP704B1*/*CYP704B2* (Wilson et al., 2001; Sorensen et al., 2003; Li et al., 2006, 2010, 2011; Morant et al., 2007; Dobritsa et al., 2009; Chen et al., 2011; Phan et al., 2011; Shi et al., 2011; Yang et al., 2014; Pan et al., 2020).

In plants, the development of sporophytic anthers and internal pollen grains is considered to be a complex biological process that is controlled by a precisely transcriptional regulatory network involving a series of genes (Feng et al., 2012). Based on mutant and interaction analysis, a number of principal genes controlling this process have been identified in *Arabidopsis*. Such genes include *DYT1*, *AMS*, *TDF1*, *MYB80*, *MS1*, *MS2*, *LAP5*, *LAP6*, *CYP703A2*, and *CYP704B1* (Wilson et al., 2001; Sorensen et al., 2003; Zhang et al., 2006; Morant et al., 2007; Zhu et al., 2008a; Dobritsa et al., 2009; Kim et al., 2010; Chen et al., 2011; Phan et al., 2011). With homologous genes characterized in rice or/and other plants, further studies implied that these genes are evolutionarily conserved. MYB80 is a key transcription factor located downstream of AMS to control the tapetum development and pollen exine formation. MYB80 was reported to directly or indirectly regulate the expression of many functional genes being responsible for the synthesis or transport of lipidic components required for anther development (Phan et al., 2011; Xiong et al., 2016; Lu et al., 2020). Genes, such as *CYP703A2*, *MS2*, *LAP5*, and *LAP6*, show a significant down-regulation in the *MYB80* mutant. Further research has found that MYB80 protein could induce the expression of these genes, thereby supporting the proposal that MYB80 acts as a pivotal connector for upstream TFs and downstream enzyme genes during anther development (Phan et al., 2011). Notably, GhMYB80 can rescue the male sterile phenotype of *AtMYB80* in *Arabidopsis* (Xu et al., 2014), indicating its potential role for anther development in cotton.

The sn-Glycerol-3-phosphate O-acyltransferase (GPAT, EC2.3.1.15) is a key enzyme responsible for the synthesis of glycerophospholipids and triacylglycerol (TAG) (Zheng et al., 2003). Previous studies revealed that diverse GPATs may catalyze the transfer of an acyl group from acyl-CoA/acyl-ACP to the different positions (sn-1 or sn-2) of glycerol-3-phosphate (G3P) (Zheng and Zou, 2001). In *Arabidopsis*, 10 GPATs (ATS1 and AtGPAT1-9) have been identified. ATS1 and AtGPAT9 possess sn-1 acyltransferase activity and they have been reported to contribute to the synthesis of storage oil (Nishida et al., 1993; Gidda et al., 2009). AtGPAT1-8 are sn-2 acyltransferases likely involved in the biosynthesis of extracellular lipids barrier polyesters, such as cutin and suberin (Yang et al., 2012). AtGPAT4 and AtGPAT8 are required for the accumulation of cutin monomers in leaves and stems (Li et al., 2007). AtGPAT5/7 predominantly contribute to the synthesis of suberin, while, AtGPAT7 has also a unique role in wounding response (Yang et al., 2012). AtGPAT1 and AtGPAT6 have been reported to be essential for pollen development and largely redundant in tapetal development. Both *gpat1* and *gpat6* mutants exhibit abnormal tapetal cells and reduced mature pollen grains, while the *gpat1 gpat6* double mutant shows short filaments and a complete male sterility (Zheng et al., 2003; Li et al., 2012). Moreover, OsGPAT3 was found to play a crucial role in anther cuticle formation and pollen exine development in rice (Men et al., 2017). Its homologous gene in maize, *ZmMS33*, also benefits male fertility (Zhang et al., 2018; Zhu et al., 2019). Despite the importance of understanding the roles of GPATs, few relative researches have been performed in cotton. To date, the GPAT family members have been identified in four species: *G. raimondii*, *G. arboreum*, *G. hirsutum*, and *G. barbadense*. Several probably storage oil-related genes have also been found (Cui et al., 2019). However, the contribution of GPAT genes in cotton male sterility has not been investigated.

In this study, the pivotal role of *GhGPAT12*/*25* in synthesizing the glycerol monomers for anther cuticle formation and pollen exine development in cotton has been investigated. GhGPAT12/25 are sn-2 glycerol-3-phosphate acyltransferases and can transfer the acyl group of palmitoyl-CoA to G3P. As paralogs pair, *GhGPAT12* and *GhGPAT25* have similar temporal expression pattern and are restricted in tapetum and pollen exine during the early anther developmental stages. Moreover, CRISPR/Cas9-mediated knockout of *GhGPAT12*/*25* caused a male sterile phenotype. Then, it was shown that GhMYB80s are likely to directly activate the expression of *GhGPAT12*/*25*. This work determined that GhGPAT12/25 participate in the conserved pathway to synthesize lipidic monomers required for anther cuticle formation and pollen exine development in cotton.

## Materials and Methods

### Plant Materials

The upland cotton TM-1 was used in this study. The plants for DNA or RNA extraction were cultivated in conventional fields of the Institute of Cotton Research, Chinese Academic Agricultural Sciences (ICR, CAAS) (Anyang, 35°120N, 113°370E). The plants for virus-induced gene silencing (VIGS) assay were cultivated in a phytotron with 16 h:8 h light:dark photoperiod.

### DNA, RNA, and cDNA

Total DNA of TM-1 anthers was extracted using the CTAB method (Porebski et al., 1997). Total RNA of various tissues and anthers of different developmental stages were isolated using the RNAprep Pure Plant Kit (Tiangen, Beijing, China). The first-strand cDNA was prepared using the PrimeScript RT reagent kit with a gDNA Eraser (Takara, Dalian, China), following the manufacturer’s instructions.

### Heterologous Expression of GhGPAT12/25 and Enzyme Assays

For heterologous expression of GhGPAT12/25 proteins, a pYES2 vector (Miaoling Bio^1^) was used for expression vector construction. The recombinant pYES2 plasmids were then transformed into yeast strain *gat1*Δ (BY4742, Matα, his3C1, leu2C0, lys2C0, ura3C0, and YKR067w:kanMX4). The induction of GPAT expression was as described by Zheng et al. (2003) with an induction time of 30 h. After centrifugation (1500 × *g* for 5 min), the cell pellets were resuspended in buffer [20 mM Tris-HCl (pH 7.9), 10 mM MgCl_2_, 1 mM EDTA, 5% (vol/vol) glycerol, 1 mM DTT, and 0.3 M ammonium sulfate]. The homogenates were prepared following the procedure established by Yang et al. (2010). The GPAT activity of GhGPAT12/25 was determined using Tissue GPAT Assay Kit (GenMed, Shanghai, China).

### qRT-PCR

qRT-PCR analyses were carried out using SYBR Premix Ex Taq (Tli RNaseH Plus) (Takara, Dalian, China). Gene-specific primers were directly obtained from qPrimerDB^2^. The cotton ACTIN 7 was used to as an internal control gene. The ΔΔCt algorithm was used for calculating relative gene expression. All primers used in this study are presented in Supplementary Table 3.

### RNA *in situ* Hybridization

TM-1 anthers at various developmental stages were sampled and fixed in formalin/acetic acid/alcohol fixative solution for 12 h at 4°C. After being dehydrated through the ethanol series, anther samples were embedded in paraffin and sectioned to a thickness of ∼7 μm using a rotary microtome (RM2016, Leica). The 29-bp specific fragment corresponding to the GhGPAT12/25 cDNA was used to design the antisense and sense probes. Digoxigenin-labeled RNA probes were labeled using DIG-UTP (Roche). Details of RNA hybridization and signal detection of the hybridized probes are described by Kouchi and Hata (1993).

### Bioinformatic Analysis of GhGPAT12/25

The DNA and CDS sequences of *GhGPAT12*/*25* were cloned from TTP stage anthers of TM-1. The basic characters of GhGPAT12/25 proteins were analyzed using ProtParam^3^. Their conserved domains were predicted using Pfam^4^. The 26 corresponding amino acid homologous sequences from 10 different species were obtained from NCBI^5^ and TAIR^6^. Multiple sequence alignment of GhGPAT12/25 and homologous sequences of other species were performed using DNAMAN. A neighbor-joining phylogenetic tree was constructed using MEGA 6.0^7^ and 1000 repetitions of bootstrap analysis were performed.

### CRISPR/Cas9-Mediated Knockout

The full-length DNA sequences of *GhGPAT12*/*25* were analyzed using CRISPR-P software^8^, and two sgRNA targets were selected to assemble into the pRGEB32-GhU6.7-NPT II expression vector. Then, the recombinant vector was transferred to *Agrobacterium tumefaciens* (GV3101). Upland cotton cv. HM-1 was used as the transformation receptor. The transgenic plants were obtained following the method described by Li et al. (2019b). The HI-TOM analysis described by Liu et al. (2019b) was used to check the sequences of transgenic plants.

### Cytological Observation

A 1% iodine/potassium iodide solution (I_2_-KI) was used to check the pollen viability. Anthers in various developmental stages of WT and mutant were sampled for paraffin section, SEM, TEM, and TUNEL. The detailed methods were as described by Uzair et al. (2020), Zhang et al. (2020).

### Wax Determination

WT and mutant anthers were collected during the mature stage. The surface areas of cotton anthers in WT and mutant were determined using microscopy. For waxes quantification, 100 mg of freeze-dried anther was submersed in 10 ml of chloroform (55°C) for 1 min. Extraction was repeated once, and the chloroform extracts were spiked with 100 μg of C24 Alkan. The solvents were evaporated under nitrogen gas. The remaining compounds were incubated with 200 μl of BSTFA (Sigma-Aldrich) and 200 μl of pyridine for 40 min at 70°C. These derivatized samples were then analyzed using GC-MS and GC-FID.

### Yeast One-Hybrid Assay

The promoter sequences of *GhGPAT12*/*25* were analyzed using New PLACE^9^ to identify specific *cis*-element motifs bound by upstream genes. Two specific promoter segments were synthesized for three copies and constructed into the pHIS2 vector. The CDS sequences of *GhDYT1-A* (*GH_A10G0179*), *GhAMS-A* (*GH_A12G0356*), *GhMYB80-A* (*GH_A04G0015*), *GhTDF1-A* (*GH_A13G1049*), and *GhbHLH91-A* (*GH_A06G1836*), potential upstream transcription factors that showed similar expression pattern with *GhGPAT12*/*25*, were cloned into the pGADT7 vector. Each pHIS2 bait vector was co-transformed with recombinant pGADT7 prey vector to Y187 yeast strain. The co-transformed yeasts were incubated on SD/-Leu/-Trp selective medium and further identified on SD/-Leu/-Trp/-His selective medium with 200 mM 3-amino-1,2,4-triazole (3-AT). The combinations of pGADT7 empty vector and recombinant pHIS2 vectors were employed as negative controls.

### Dual-Luciferase Assay

A transient dual-luciferase assay in tobacco leaves was carried out to confirm the activation effect of GhMYB80s on *GhGPAT12*/*25*. The promoter sequence of *GhGPAT12* was amplified and cloned into the pGreenII0800-LUC vector as the reporter plasmid. The CDS of *GhMYB80*-*A* was cloned into the pGreenII 62-SK vector as effecter plasmid. The recombinant vectors were then transformed into the GV3101 (pSoup-p19) strain. Subsequently, equal *A. tumefaciens* cells that carried the reporter and effector vectors were co-infiltrated into tobacco leaves. Two days later, the infected tobacco leaves were sampled for LUC and REN luciferase activity analysis. To achieve this, the Dual-Luciferase Reporter Assay System (Promega, Madison, WI, United States) and GloMax 20/20 Luminometer (Promega) were used. Moreover, the pGreenII0800-LUC-pGhGPAT12 and pGreenII 62-SK (empty) vectors were used as a negative control, and each combination was replicated six times.

### VIGS Assay

To further illustrate the potential function of GhMYB80 in cotton anther development, a VIGS assay was performed using upland cotton TM-1 as receptor. Briefly, a 300-bp fragment of the *GhMYB80-A* CDS located outside of the conserved region was cloned and constructed into the pCLCrVA vector. The specific primers are listed in Supplementary Table 3. The empty pCLCrVA and pCLCrVA-*PDS* were used as negative and positive controls, respectively. These plasmids were transferred into *A. tumefaciens* (strain GV3101). The *A. tumefaciens* cultures containing the helper vector (pCLCrVB) and pCLCrVA or its derivative (pCLCrVA-*PDS* and pCLCrVA-*GhMYB80s*) vectors were mixed at ratio of 1:1 and then co-injected into the fully expanded cotyledons of *c*. 10-day-old TM-1 seedlings. The plants were cultivated at a 23°C phytotron with 16 h:8 h light:dark photoperiod. At the flowering stage, the phenotype of pCLCrVA-*GhMYB80s* individuals were observed for the identification of male sterile plants, and the TTP stage anthers of different plants were collected for qRT-PCR analysis.

### RNA-Seq

A total of eight libraries, comprising two cotton groups (*ghgpat12*/*25* and WT) at two stages (TTP and early UNP stage) and two biological replicates, were sequenced using Illumina NovaSeq 6000 to generate paired-end reads. Low-quality reads were first removed, and the resulting clean reads were aligned with the *G. hirsutum* (TM-1) genome (Hu et al., 2019). StringTie was used to accurately quantify the fragments per kilobase of transcript per million mapped reads (FPKM) for calculating gene expression levels. Differential expression genes were obtained using the DESeq R package. Genes with the | log2 fold change| ≥ 1 and FDR ≤ 0.01 were considered to be differentially expressed. Gene Ontology (GO) enrichment analysis of DEGs was performed using OmicShare tools^10^ and the ClueGO plugin in Cytoscape 3.3.0. STRING^11^ was used to identify the known and predicted interactions in DEGs. Then, the ClueGO plugin was used for the enrichment analysis of PPI network-containing genes. RNA-seq raw data are accessible in the NCBI Sequence Read Archive(SRA) database under Accession Number PRJNA698752.

## Results

### *GhGPAT12*/*25* Have Early Anther Stage-Specific Expression in Cotton

**FIGURE 1:**
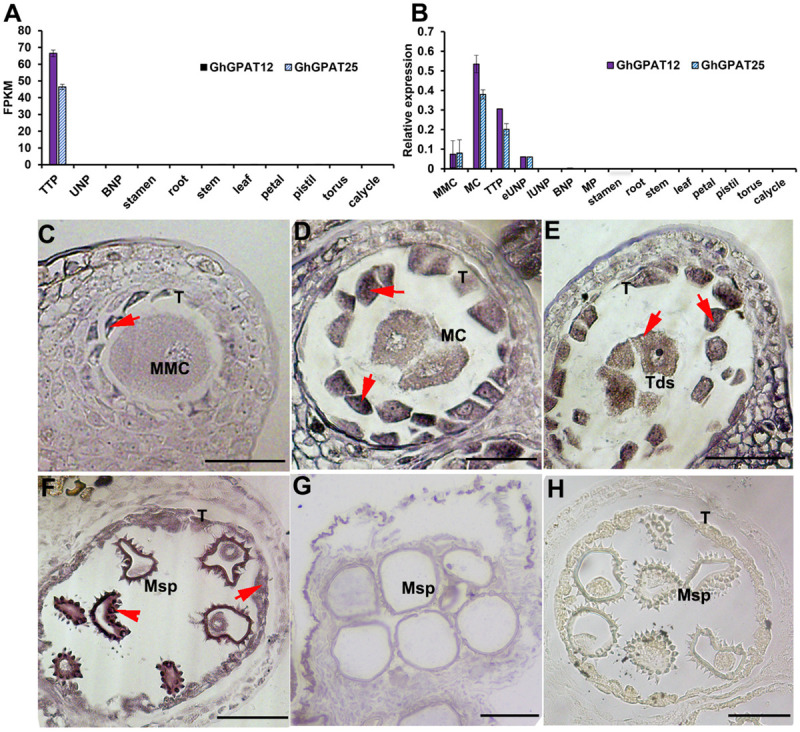
Expression pattern of *GhGPAT12*/*25*. **(A)** Expression levels of *GhGPAT12*/*25* based on RNA-seq data. Error bars indicate ± S.D. (*n* = 3 biological replicates). **(B)** qRT-PCR analysis of *GhGPAT12*/*25* in different developmental stage anthers and other tissues. Error bars indicate ± S.D. (*n* = 3 biological replicates). **(C–G)** RNA *in situ* hybridization assay of *GhGPAT12*/*25* in WT anthers of panel **(C)** microspore mother cell (MMC) stage, **(D)** meiosis cell (MC) stage, **(E)** tetrad pollen (TTP) stage, **(F)** uninucleate pollen (UNP) stage, and **(G)** binucleate pollen (BNP) stage. **(H)** Anther of uninucleate pollen (UNP) stage hybridized with sense probe. MMC, microspore mother cell; MC, meiosis cell; Msp, microspore; T, tapetum; Tds, tetrads. The red arrows show positive signal in anthers. Bar = 50 μm.

Previous RNA-seq data of anther samples at the tetrad pollen (TTP), uninucleate pollen (UNP), and binucleate pollen (BNP) stages are important references to identify crucial genes for anther development (Zhang et al., 2020). Public expression data provide an additional rich resource of information on multiple tissues expression of cotton genes (Zhang et al., 2015). Based on the combination analysis of these data, *GhGPAT12*/*25* (glycerol-3-phosphate acyltransferase genes located on A12/D12 chromosomes) were found expressed in anthers but not in any other tissues, and they also showed strict expression during TTP stage (Figure 1A).

To confirm this, qRT-PCR analysis was performed using anthers at the developmental stages of microspore mother cell (MMC), meiosis cell (MC), tetrad pollen (TTP), early uninucleate pollen (eUNP), late uninucleate pollen (lUNP), binucleate pollen (BNP), mature pollen (MP), and vegetative tissue samples of cotton plants. The results suggested that *GhGPAT12*/*25* were anther-specific expressed and showed negligible expression in all the other tissues tested. In anther tissues, the expression of *GhGPAT12*/*25* was detectable as early as the MMC stage, peaked at the MC stage, declined gradually at the TTP stage, and showed a lower level after the eUNP stage. In addition, *GhGPAT12* showed a similar expression pattern to *GhGPAT25*, but its expression levels were higher at MC and TTP stages than those of *GhGPAT25* (Figure 1B).

RNA *in situ* hybridization with anther sections was performed to further precisely determine the spatial and temporal patterns of *GhGPAT12*/*25* transcripts. *GhGPAT12*/*25* signal was initially detected in tapetal layer at the MMC stage (Figure 1C), and then a strong expression signal was detected predominantly in the tapetum and microspores from the MC to the TTP stage (Figures 1D,E). From the TTP stage onward, the signal of *GhGPAT12*/*25* transcripts decreased in the tapetum but showed relatively high abundance in microspores exine, especially on the spines (Figure 1F). Subsequently, the signal was undetectable in the BNP stage (Figure 1G). Meanwhile, no expression signals were detected by the sense probe in anthers sections during the UNP stage (Figure 1H). This expression pattern implied that *GhGPAT12*/*25* may play specific roles in microspore development and pollen wall formation.

### GhGPAT12/25 Are Active sn-2 Glycerol-3-Phosphate Acyltransferases Conserved in Land Plants

**FIGURE 2:**
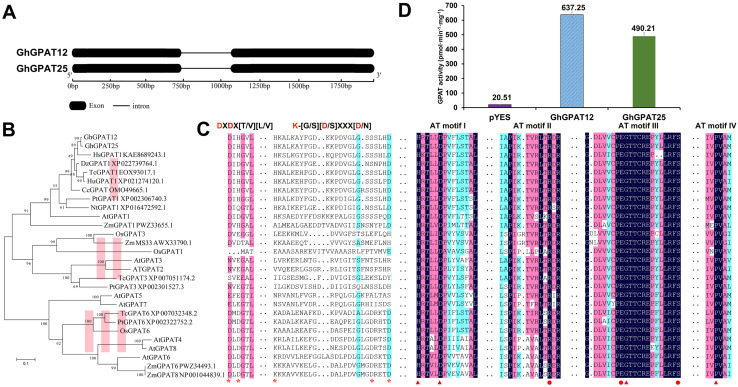
Gene and protein characterization of GhGPAT12/25. **(A)** Structure of *GhGPAT12*/*25* genes. Black boxes and lines indicate exons and introns, respectively. **(B)** Phylogenetic tree analysis of GhGPAT12/25 and their homologous sequences from other species. **(C)** Multiple sequence alignment of GPAT amino acids from various species. DXDX[T/V][L/V] and K-[G/S][D/S]XXX[D/N] are important motif in the haloacid dehalogenase (HAD)-like domain. AT motifs I–IV are conserved regions in acyltransferase domain. Asterisks indicate critical sites in the HAD-like domain that are indispensable for phosphatase activity of GPATs. Binding and catalytic residues in acyltransferase domain are marked by dots and triangles, respectively. **(D)** GPAT activity in yeast homogenates of *gat-1*Δ mutants transformed with either the empty pYES2 vector or the recombined plasmids containing the *GhGPAT12*/*25* genes. Error bars indicate ± S.D. (*n* = 3 biological replicates).

*GhGPAT12*/*25* were cloned from the TTP stage anther DNA/cDNA of upland cotton TM-1. The genomic DNA sequence was 1976/1977 bp in length, containing two exons and one intron (Figure 2A). The coding region was 1626 bp, and the corresponding 541-aa protein had a molecular mass of 60.99/60.98 kDa and an isoelectric point of 9.12/9.29.

GhGPAT12/25 were glycerol-3-phosphate acyltransferases and were predicted to contain a conserved acyltransferase domain (Pfam: PF01553) at the C-terminal region. To understand the evolutionary roles of GhGPAT12/25 and gain information on their potential function, the full-length proteins were used as queries to search their homologs in two public databases: National Center for Biotechnology Information (NCBI see text footnote 5) and The *Arabidopsis* Information Resource (TAIR see text footnote 6). This search yielded a total of 26 close homolog protein sequences from 10 different species. Subsequently, a phylogenetic tree was constructed, and the 28 sequences were grouped into three main clades (Figure 2B). AtGPAT1–8 were reported to belong to sn-2 GPATs, which contribute to the formation of polyester, such as cutin and suberin (Yang et al., 2012). The distribution of these *Arabidopsis* members in three different groups of the phylogenetic tree indicated that homologous proteins in these different species may have similar functions with AtGPAT1–8.

GhGPAT12/25, together with their orthologs from *Hibiscus syriacus*, *Durio zibethinus*, *Theobroma cacao*, *Herrania umbratical*, *Corchorus capsularis*, *Populus trichocarpa*, *Nicotiana tabacum*, *Zea mays*, and *Arabidopsis*, were grouped in clade I. OsGPAT1 and all GPAT2/3 proteins were clustered in clade II, while clade III included the remaining sequences (Figure 2B). Sequence alignment showed that GhGPAT12 and GhGPAT25 share an acyltransferase (AT) domain with similar conserved boxes (AT-1 to AT-IV) containing the corresponding catalytic residues and binding residues with 26 other proteins (Figure 2C). With the exception of the AT region, haloacid dehalogenase (HAD)-like domains containing phosphohydrolases were recognized at the N-terminal region of GPAT. According to the protein sequence analysis, except for AtGPAT5 and AtGPAT7, other proteins of clade III had the typical DXD signature and GDXXXD motif required for phosphatase activity (Figure 2C). However, other 20 proteins lacked these intact residues (Figure 2C), indicating their absent phosphatase activity. Furthermore, members of clade III, such as AtGPAT4–8, were involved in the formation of polyester in vegetative organs and the improvement of tolerance to environment stresses (Yang et al., 2012). Proteins in clade I or II, like AtGPAT1, OsGPAT3, and ZmMS33, have been shown to be essential for pollen development (Zheng et al., 2003; Men et al., 2017; Zhang et al., 2018; Zhu et al., 2019; Figure 2C). These analyses suggested the functional differentiation of genes from different clades in vegetative and reproductive development. Altogether, these observations indicated a conserved structure of GhGPAT12/25 in sn-2 glycerol-3-phosphate acyltransferase and potential roles in plant male reproductive development.

To determine whether GhGPAT12/25 have the activity to acylate glycerol-3-phosphate, *GhGPAT12* and *GhGPAT25* were cloned into pYES2 vector; both proteins were heterologously expressed in the yeast strain *gat1*Δ, which harbors a mutant ER-bound *GPAT* gene with very low GPAT activity (Zheng and Zou, 2001). Under the control of the galactose-inducible promoter of *GAL1*, the *GhGPAT12* and *GhGPAT25* proteins were expressed, and total yeast homogenates were prepared for the detection of glycerol-3-phosphate acyltransferase activities with palmitoyl-CoA as the fatty acyl donor. Thus, overexpression of *GhGPAT12* and *GhGPAT25* resulted in an increase of about 617 and 470 pmol⋅min^–1^⋅mg^–1^ in glycerol-3-phosphate acyltransferase activities, respectively (Figure 2D). The significant increases of specific activities in yeast proved directly that *GhGPAT12* and *GhGPAT25* indeed encoded active glycerol-3-phosphate acyltransferases.

### CRISPR/Cas9-Mediated Knockout of *GhGPAT12*/*25* Leads to Male Sterility

**FIGURE 3:**
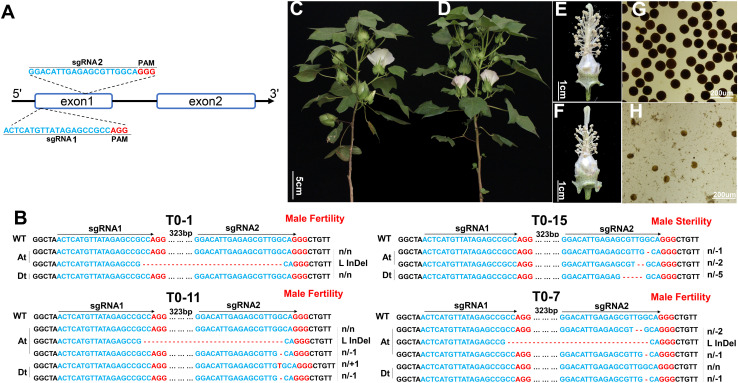
Functional characterization of GhGPAT12/25 by CRISPR/Cas9-mediated knockout assay. **(A)** Gene structure of *GhGPAT12*/*25* and the target sites of sgRNAs in exon1. The GGG and AGG in red represent the PAM motifs. **(B)** Variation information of transgenic lines T_0_-1, 7, 11, and 15. The PAM sequence is shown in red. Deletions are denoted with red dashes. Insertions are shown as red letters. The mutation types are shown on the right. L InDel indicates large InDel. **(C–H)** Phenotypic characteristics of *ghgpat12*/*25* and its wild-type HM-1. **(C)** Adult plant, **(E)** flower, and **(G)** pollen phenotypes of the wild-type HM-1; **(D)** adult plant, **(F)** flower, and **(H)** pollen phenotypes of *ghgpat12*/*25*.

CRISPR-Cas9 was performed in upland cotton “HM-1” to knock out *GhGPAT12*/*25* genes in order to validate their role in reproductive development. Because of the high similarity of sequence and temporal expression pattern of *GhGPAT12*/*25* (Figure 1 and Supplementary Figure 1), both genes were targeted for knockout. Two sgRNAs were designed targeting the 1st exons (323 bp away from each other) and cloned into a single Cas9-sgRNA cassette (Figure 3A).

Eleven independent T_0_ transgenic lines were acquired. Hi-TOM (Liu et al., 2019b) was performed to evaluate the editing efficiency and to precisely analyze the nucleotide insertion and deletion of *GhGPAT12*/*25*. The results showed that all 11 transgenic lines were positive plants with various types of genome editing events precisely occurring at the examined gene, *GhGPAT12* or/and *GhGPAT25* (Figure 3B and Supplementary Figure 2). Among such transgenic lines, T_0_-2, T_0_-12, T_0_-15, T_0_-23, T_0_-25, and T_0_-26 possessed both edited *GhGPAT12* and *GhGPAT25* sequences at the target site, but no normal WT sequences (Figure 3B and Supplementary Figure 2). For instance, T_0_-23 exhibited three types of indels at two target sites in *GhGPAT12* and two types of indels at sgRNA2 site in *GhGPAT25* (Supplementary Figure 2). However, T_0_-1, T_0_-6, T_0_-7, T_0_-11, and T_0_-28 were incompletely edited lines that contained WT sequences in either *GhGPAT12* or *GhGPAT25* (Figure 3B and Supplementary Figure 2). Among these, the T_0_-1 and T_0_-28 plants presented WT sequences of both *GhGPAT12* and *GhGPAT25*, while the T_0_-6 and T_0_-11 plants possessed several types of indels on At and Dt sub-genes and held WT sequences of *GhGPAT12* (Figure 3B and Supplementary Figure 2). T_0_-7 showed a completely edited *GhGPAT12* gene with three types of indels, but incomplete *GhGPAT25* with one type of indel and the WT sequences (Figure 3B).

To evaluate the function of *GhGPAT12*/*25*, the T_0_ and T_2_ (F_1_ self-pollinated progeny of T_0_ × HM-1) transgenic lines were planted in agronomic field for morphological analysis. The results showed that the incompletely edited lines T_0_-1, T_0_-6, T_0_-7, T_0_-11, and T_0_-28 had a normal phenotype with fertile anthers. Whereas the completely edited lines, such as T_0_-15, exhibited normal vegetative growth similar to HM-1 (Figures 3C,D), the reproductive organs were defective (Figure 3F). The flower of T_0_-15 displayed shorter filaments with shriveled anthers (Figure 3F). The anthers did not dehisce and lacked mature pollen grains at the late developmental stages when stained with 1% I_2_-KI solution (Figures 3F,H). T_2_ plants of T_0_-15 showed fertility segregation; according to the results of the Hi-TOM analysis, the F_2_ progenies with homozygous or biallelic mutations of *GhGPAT12*/*25* were completely male sterile (Supplementary Figure 3), while the genotypes of fertile plants were homozygous wild type or heterozygous (Supplementary Figure 3). These results clearly indicated that *GhGPAT12* and *GhGPAT25* play key roles in the normal development of male organs in cotton and appear to be functionally redundant.

### GhGPAT12/25 Contribute to the Assembly of Anther Cuticle and Pollen Exine

**FIGURE 4:**
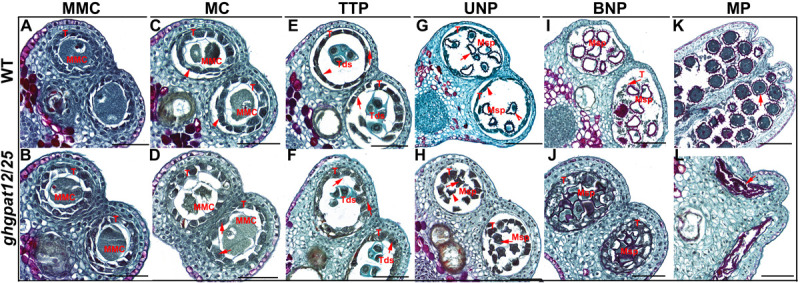
Paraffin section of anthers in different developmental stages of *ghgpat12*/*25* and HM-1. Transverse section images of HM-1 anthers shown in panels **(A,C,E,G,I,K)** and those for *ghgpat12*/*25* anthers shown in panels **(B,D,F,H,J,L)**. BNP, binucleate pollen; MMC, microspore mother cell; MC, meiosis cell; MP, mature pollen; Msp, microspore; T, tapetum; Tds, tetrads; TTP, tetrad pollen; UNP, uninucleate pollen. The red arrows show main differences in anthers. Bar = 100 μm.

To investigate the cytological effects of GhGPAT12/25, histological transverse section analysis of WT and *ghgpat12*/*25* (a T_2_ male sterile plant) anthers was employed. No significant differences between *ghgpat12*/*25* and WT were detected at the MMC stage. Their anthers formed four typical anther wall layers and their microsporocytes were located at the center of each anther locule (Figures 4A,B).

Subsequently, *ghgpat12*/*25* anthers displayed clear morphological abnormalities. At the MC stage, WT tapetal cells started to shrink and showed deep staining with toluidine blue (Figure 4C), while the *ghgpat12*/*25* tapetal cells did not appear to condense and were weakly stained (Figure 4D). At the TTP stage, anther wall layers of WT anthers became thinner, tetrads of haploid microspores were formed in anther locule, and the tapetal layer was more condensed (Figure 4E). In contrast, the wall layers of *ghgpat12*/*25* anthers seemed to be irregular and thicker, the microspores in *ghgpat12*/*25* tetrads exhibited an irregular shape, and the tapetal layer was swollen and lightly stained (Figure 4F). At the UNP stage, WT microspores covered with spines were released from the tetrads. The tapetal layer underwent PCD and apparently decayed into a thinner layer (Figure 4G). However, the microspores of *ghgpat12*/*25* showed an anomalous appearance without spines (Figure 4H). Meanwhile, the *ghgpat12*/*25* tapetum was still pachytic (Figure 4H). At the BNP stage, the WT anther locules continued to expand with thinner wall layers, microspores became spherical with large vacuoles, and the tapetum was almost completely degraded into cellular debris (Figure 4I). By contrast, *ghgpat12*/*25* displayed narrow anther locules with thicker wall layers and tapetum, as well as microspores without large vacuoles and abnormal appearance despite the presence of the expansion (Figure 4J). From the vacuolated pollen stage to the mature pollen stage, the tapetum of the WT gradually degenerated and vacuolated microspores turned to mature pollen grains with fully accumulated nutrients (Figures 4I,K). However, the tapetum of *ghgpat12*/*25* seemed to undergo acute and abnormal degradation, leaving only aborted pollen grains in the atrophic anther locules (Figures 4J,L).

**FIGURE 5:**
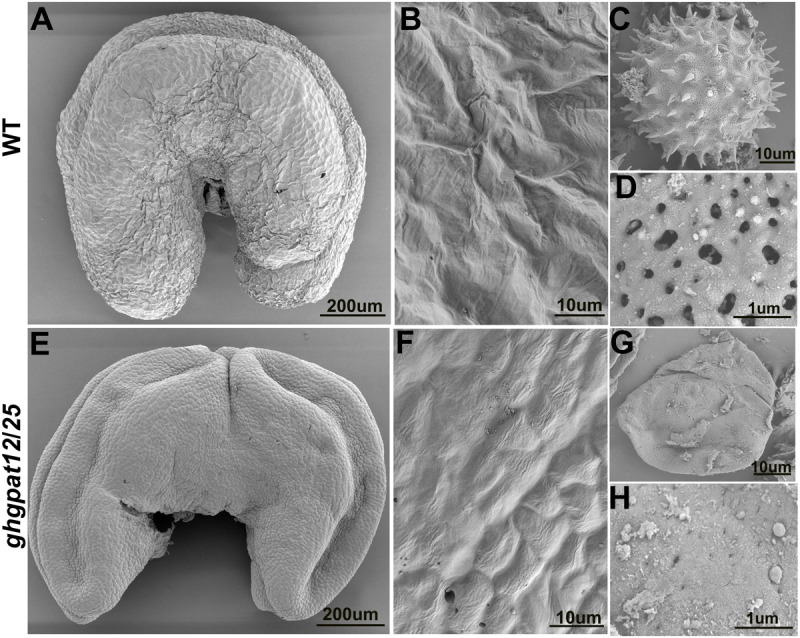
Scanning electron microscope (SEM) analysis of the anther surfaces and pollen grains in HM-1 and *ghgpat12*/*25*. Anthers of panels **(A)** HM-1 and **(E)** *ghgpat12*/*2*5 at maturation stage. The enlarged detailed view of the anther surfaces of panels **(B)** HM-1 and **(F)** *ghgpat12*/*25*. Pollen grain of panels **(C)** HM-1 and **(G)** *ghgpat12*/*25* at maturation stage. The enlarged detailed views of the pollen surfaces of panels **(D)** HM-1 and **(H)** *ghgpat12*/*25*.

Scanning electron microscopy (SEM) was used to further investigate the morphological differences of anthers and pollen grains in WT and *ghgpat12*/*25* at the late development stages. The WT anther exhibited a plump and normal cuticle-covered surface (Figures 5A,B). The pollen grains were covered by exine pattern full of spines and apertures (Figures 5C,D). In *ghgpat12*/*25*, a shriveled and relatively smooth anther epidermis (Figures 5E,F) and shrunken pollen grains were produced as a result of the disorganized cuticle structures and the anomalously assembled exine (Figures 5G,H).

**FIGURE 6:**
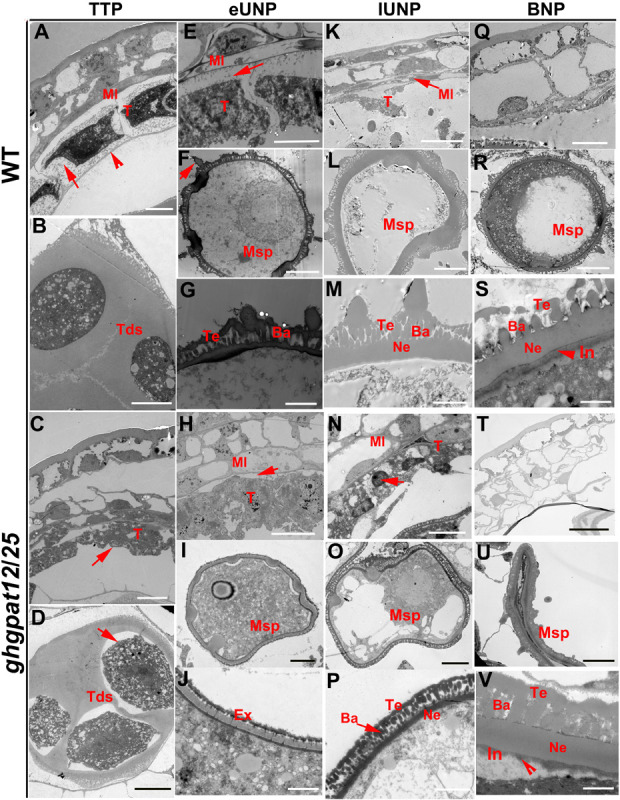
Transmission electron microscopy (TEM) analysis of anthers in HM-1 and *ghgpat12*/*25*. **(A,E,K,Q)** Cross sections of the HM-1 anther cell wall at the **(A)** TTP stage, **(E)** eUNP stage, **(K)** lUNP stage, and **(Q)** BNP stage. **(C,H,N,T)** Cross sections of the *ghgpat12*/*25* anther cell wall at the **(C)** TTP stage, **(H)** eUNP stage, **(N)** lUNP stage, and **(T)** BNP stage. **(B,D)** Tds of panel **(B)** HM-1 and **(D)** *ghgpat12*/*25*. **(F,L,R)** Pollen grains of HM-1 at the **(F)** eUNP stage, **(L)** lUNP stage, and (R) BNP stage. **(I,O,U)** Pollen grains of *ghgpat12*/*25* at the **(I)** eUNP stage, **(O)** lUNP stage, and **(U)** BNP stage. **(G,M,S)** Pollen exine of HM-1 at the **(G)** eUNP stage, **(M)** lUNP stage, and **(S)** BNP stage. **(J,P,V)** Pollen grains of *ghgpat12*/*25* at **(J)** eUNP stage, **(P)** lUNP stage, and **(V)** BNP stage. Ba, bacula; Ex, exine; In, intine; Ml, middle layer; Msp, microspore; Ne, nexine; T, tapetum; Tds, tetrads; Te, tectum. The red arrows show main differences between HM-1 and *ghgpat12*/*25* in tapetum, tetrads, pollen grains and exine. Bars: 10 μm in panels **(A-C,F,I)** and **(L-N,Q,T)**, 20 μm in panels **(D,G,J)** and **(O,R,U)**, and 2 μm in panels **(E,H,K,P,S,V)**.

To more precisely characterize the defects in *ghgpat12*/*25*, transmission electron microscopy (TEM) analysis was performed on the anthers from the TTP stage to the BNP stage. At the TTP stage, the tapetum showed a thin cell wall layer, the tapetal cells became largely vacuolated, and the middle layer began to degenerate (Figure 6A). Additionally, rounded microspores were observed within the tetrad (Figure 6B). Unlike those tapetum of WT plants, *ghgpat12*/*25* tapetum was disordered and its middle layer was much thicker (Figure 6C). At the same stage, although the tetrads of *ghgpat12*/*25* were also covered by callose, the microspores seemed to develop abnormally with irregular surface (Figure 6D). At the early UNP stage and within the tapetal cells of WT, the vacuoles were reabsorbed and the tapetum started to degenerate (Figure 6E). Meanwhile, the elementary structure of the exine, composed of the well-organized tectum, bacula, and nexine, was observed at the surface of the WT microspores being released from the tetrads (Figures 6F,G). Conversely, in the *ghgpat12*/*25* anthers, the middle layer persisted and became largely vacuolated (Figure 6H); the tapetal cells seemed slightly stained and became abnormally expanded, which caused one side to be in direct contact with the middle layer (Figure 6H). The *ghgpat12*/*25* microspores displayed an irregular shape (Figure 6I), and the pollen exine seemed to contain relatively normal nexine, but the tectum and bacula were poorly developed and lacked the assembly of spines (Figure 6J). At the late UNP stage, the WT microspore underwent large vacuolation period, the tapetum continued to degenerate, and the sporopollenin was deposited onto the microspore surface thickening the exine (Figures 6K–M). However, a stunted middle layer and swollen tapetum cells with intact nuclei were observed in the *ghgpat12*/*25* anther (Figure 6N). The *ghgpat12*/*25* pollen nexine did not show significant thickening, and the assembly of sexine was still abnormal (Figures 6O,P). At the BNP stage, the WT anther showed an almost completely degenerated tapetal cell layer (Figure 6Q) and spherical pollen grains with accumulated starch and lipidic materials (Figure 6R). Furthermore, the pectocellulosic intine started to develop beneath the exine, and the tryphine was deposited onto the pollen wall (Figure 6S). However, *ghgpat12*/*25* anther showed no obvious degeneration of the middle layer but a vanished tapetal layer (Figure 6T), leaving aborted microspores with thin intine and absent tryphine in anther locules (Figures 6U,V).

In synthesis, it can be concluded that GhGPAT12/25 are required for the development of an anther cell layer, tapetum PCD, pollen wall assembly, and anther cuticle formation.

### DNA Fragmentation in *ghgpat12*/*25* Tapetal Cells Is Abnormal

**FIGURE 7:**
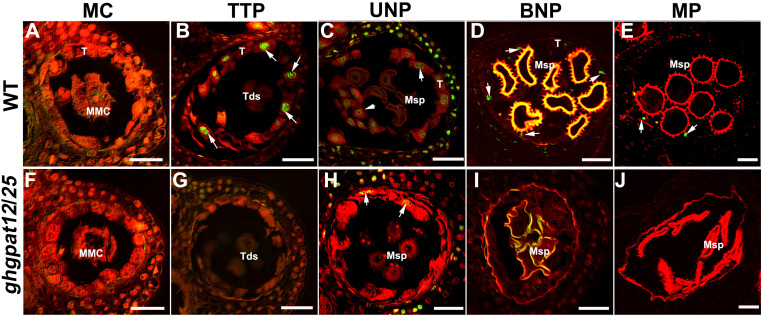
TUNEL assay for the detection of DNA fragmentation in anthers of HM-1 and *ghgpat12*/*25*. **(A–E)** DNA fragmentation in HM-1 anthers at the **(A)** meiosis cell (MC) stage, **(B)** tetrad pollen (TTP) stage, **(C)** uninucleate pollen (UNP) stage, **(D)** binucleate pollen (BNP) stage, and **(E)** mature pollen (MP) stage. **(F–J)** DNA fragmentation in *ghgpat12*/*25* anthers at the **(F)** meiosis cell (MC) stage, (G) tetrad pollen (TTP) stage, **(H)** uninucleate pollen (UNP) stage, **(I)** binucleate pollen (BNP) stage, and **(J)** mature pollen (MP) stage. Msp, microspore; T, tapetum; Tds, tetrads. The white arrows show positive signal in anthers. Bar = 20 μm.

The timely degradation of tapetal cells caused by the process of PCD is considered to be vital for anther cuticle formation and pollen development. Paraffin section observation and TEM suggested that *ghgpat12*/*25* tapetal cells abnormally expanded with the development of pollen. To examine whether *ghgpat12*/*25* anthers are defective in the tapetum PCD process, a TUNEL (terminal deoxynucleotidyl transferase-mediated dUTP nick-end labeling) assay was performed. DNA fragmentation was not detected at the MC stage in both WT and *ghgpat12*/*25* tapetal. Tapetal DNA fragmentation signals were firstly visualized in the WT anthers at the TTP stage (Figures 7A,F). As the tapetum layer continued to degenerate, positive TUNEL signals continued to increase until reaching a maximum at the UNP stage (Figure 7B), and then the signals declined and almost disappeared after the BNP stage (Figures 7C,D). However, no clear DNA fragmentation was detected in *ghgpat12*/*25* anthers, with the exception of the few signals scattered across the tapetum at the UNP stage (Figures 7G–J). These TUNEL assay results further indicated that the precise PCD of *ghgpat12*/*25* tapetum was disrupted.

### Loss of Function of *GhGPAT12*/*25* Affects the Expression of Genes Responsible for Lipid Synthesis and Transport

**FIGURE 8:**
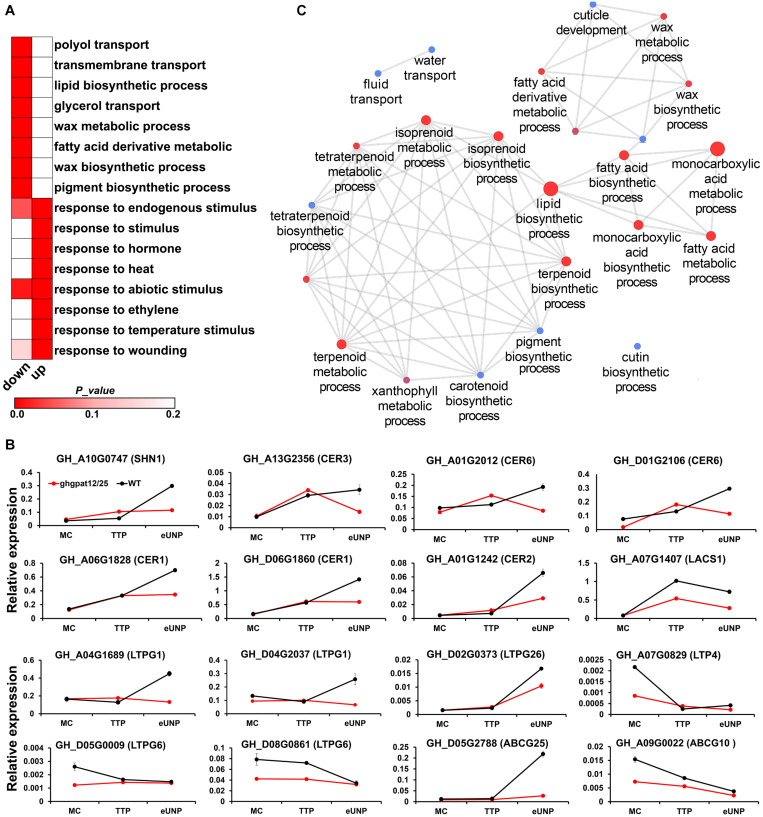
RNA-seq and qRT-PCR analysis of anthers in HM-1 and *ghgpat12*/*25*. **(A)** Gene Ontology (GO) analysis of down-regulated and up-regulated DEGs. **(B)** qRT-PCR analysis of DEGs related to the synthesis and transport of lipidic monomers required for cuticle formation. **(C)** Biological process enrichment analysis of PPI DEGs by ClueGO plug-in in Cytoscape. The size of the nodes represents the number of genes. Node color, from blue to red, indicates increase in significance of biological processes. Error bars indicate ± S.D. (*n* = 3 biological replicates).

To further gain insight into the functional mechanism of *GhGPAT12*/*25* in the control of anther development, RNA-seq of anthers at the TTP and early UNP stage was established to compare the global gene expression of *ghgpat12*/*25* and WT plant. A total of 3655 original differentially expressed genes (DEGs) were filtered with the | log_2_ fold change| ≥ 1 and FDR ≤ 0.01. Of these genes, 1111 were down-regulated (uninvolved in up-regulation in any stages) and 2315 were up-regulated (uninvolved in down-regulation in any stages). Enrichment analysis of DEGs showed that the down-regulated genes were involved in “polyol transport,” “transmembrane transport,” “lipid biosynthetic process,” “glycerol transport,” and “wax metabolic process,” among others (Figure 8A), whereas the up-regulated genes were mainly enriched (Figure 8A) to be responsive to various stimuli, such as hormone, chemical, chitin, etc.

Notably, the down-regulated genes included *CER1* (*GH_A06G1828* and *GH_D06G1860*), *CER3* (*GH_A13G2356*), and *CER6* (*GH_A01G2012* and *GH_D01G2106*) (Supplementary Table 1), which have been reported to be male sterile genes contributing to the biosynthesis of cuticle membrane and wax in *Arabidopsis* (Aarts et al., 1995; Fiebig et al., 2000; Ariizumi et al., 2003). Moreover, other wax and cuticle pathway involving genes, such as *LACS1* (Lu et al., 2009) (*GH_A07G1407*), *CER2* (Haslam et al., 2012) (*GH_A01G1242*), and *SHN1* (Broun et al., 2004) (*GH_A10G0747*), also exhibited lower expression in *ghgpat12*/*25* (Supplementary Table 1). For the assembly of anther cuticle, lipidic monomers synthesized by glycerol-3-phosphate acyltransferase are required to be transferred to the epidermis layer by LTPs and ABC transporter proteins (ABCGs) (Li-Beisson et al., 2013). Here, many *LTPs* and *ABCGs* genes were found to be down-regulated in the mutant (Supplementary Table 1); among such genes are *LTPG1* (*GH_A04G1689* and *GH_D04G2037*), *LTPG6* (*GH_D05G0009* and *GH_D08G0861*), *LTP4* (*GH_A07G0829*), *LTPG26* (*GH_D02G0373*), *ABCG14* (*GH_D08G1774*), and *ABCG25* (*GH_D05G2788*). Subsequently, the decreased expression of these genes using quantitative real-time (qRT)-PCR was confirmed (Figure 8B). To further elucidate the potential regulatory mechanism of *GhGPAT12*/*25*, the known and predicted interactions among down-regulated genes were investigated, and genes with high connectivity with others in the protein–protein interaction (PPI) network were selected for functional analysis. Such investigation showed that these genes were also involved in lipid biosynthetic, wax metabolic, and cutin metabolic processes (Figure 8C), which correlated well with the GO enrichment of all down-regulated genes. Moreover, genes contributing to the biosynthetic processes of several secondary metabolites (isoprenoid, terpenoid and pigment) were also enriched in this network (Figure 8C). These results implied that the mutation of *GhGPAT12*/*25* might affect the biosynthesis and transportation of lipidic components required for the formation of exine and anther cuticle.

### Anther Cuticular Waxes Were Altered in *ghgpat12*/*25* Anthers

**FIGURE 9:**
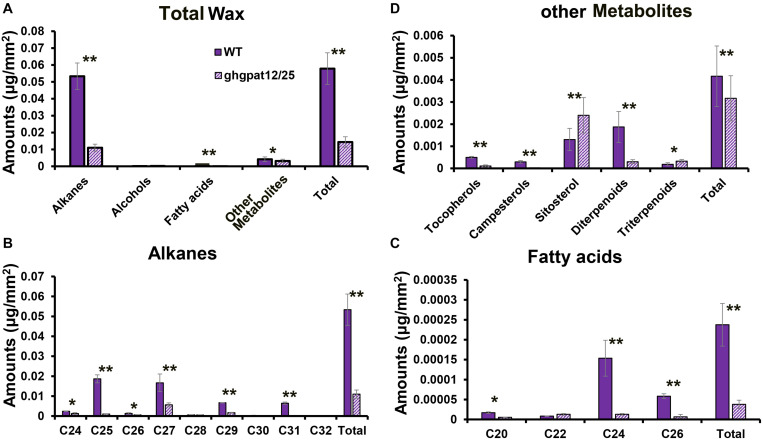
Analysis of anther waxes compositions in the anthers of WT and *ghgpat12*/*25*. **(A)** Total wax, **(B)** alkanes, **(C)** fatty acids, and **(D)** other metabolites’ amounts per unit surface area (μg/mm^2^) in WT and *ghgpat12*/*25* anthers. Student’s *t*-test, *P < 0.05 and **P < 0.01. Error bars indicate ± S.D. (*n* = 3 biological replicates).

Due to the defects of anther cuticle and pollen exine (Figures 5, 6), as well as the down-regulation of wax synthesis genes in *ghgpat12*/*25* (Figure 8B), it can be speculated that the wax enrichment in *ghgpat12*/*25* anthers was abnormal. To test this hypothesis, the compositions and quantity of cuticle waxes of the wild type and the *ghgpat12*/*25* anthers were determined by gas chromatography–mass spectrometry (GC-MS) and gas chromatography–flame ionization detection (GC-FID). After clarifying the ratio between surface area and weight of anthers in WT and *ghgpat12*/*25* (Supplementary Figure 5), the wax compositions per unit area (mm^2^) were quantified. Alkanes were determined as the major wax monomers in WT and *ghgpat12*/*25* anthers (Figure 9A). Approximately, a total of 0.058 μg waxes per mm^2^ were detected in WT anthers. However, the waxes in *ghgpat12*/*25* anthers (0.014 μg/mm^2^) were 75% less than those in the WT anthers (Figure 9A and Supplementary Table 2). The affected waxes in *ghgpat12*/*25* anthers were attributed to the reduction of alkanes (C24, C25, C26, C27, C29, C30, C31, and C32) and fatty acids (C20, C24, and C26) (Figures 9B,C and Supplementary Table 2). Additionally, a significant decrease in the levels of other metabolites, like tocopherols, campesterols, and diterpenoids, was also observed in *ghgpat12*/*25* anthers (Figure 9D and Supplementary Table 2). On the other hand, the contents of alcohols (C22, C24, and C26) in the anthers of WT and *ghgpat12*/*25* were not significantly different (Supplementary Figure 6 and Supplementary Table 2). These data implied that the loss of function of *GhGPAT12*/*25* can affect the enrichment of wax monomers in cotton anthers.

### *GhGPAT12*/*25* Were Activated by GhMYB80

**FIGURE 10:**
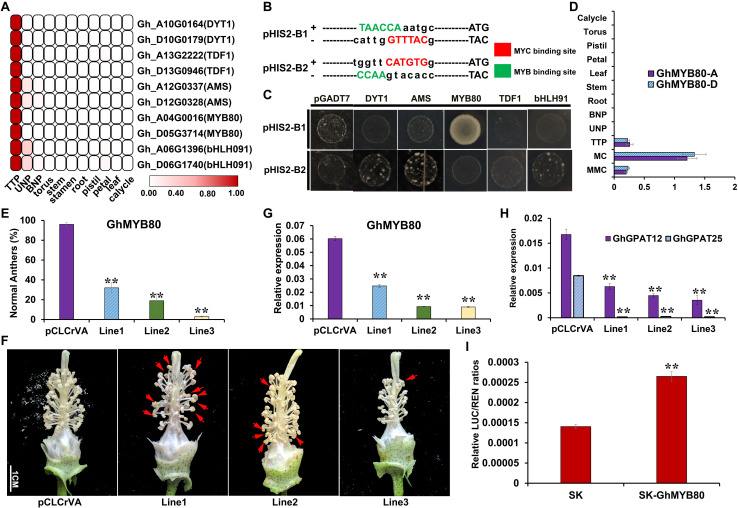
Regulatory relationship between *GhGPAT12*/*25* and GhMYB80. **(A)** Transcription factors showing similar expression pattern with *GhGPAT12*/*25*. **(B)** Binding site sequences of MYB and bHLH (MYC) TFs on the promoters of *GhGPAT12*/*25*. The binding sites of MYB and bHLH (MYC) are located on the complementary strands. **(C)** Interaction between upstream TFs and the probably targeting promoter sequences of *GhGPAT12*/*25* in yeast one-hybrid assay. **(D)** Expression pattern of *GhMYB80s*. **(E)** The proportion of normal anthers in pCLCrVA and pCLCrVA-*GhMYB80s* lines. **(F)** Flower phenotype of pCLCrVA and pCLCrVA-*GhMYB80s* lines. The red arrows show normal anthers. **(G)** Relative expression levels of *GhMYB80s* in pCLCrVA and pCLCrVA-*GhMYB80s* lines. **(H)** Relative expression levels of *GhGPAT12*/*25* in pCLCrVA and pCLCrVA-*GhMYB80s* lines. **(I)** The relative LUC/REN ratios of the control and test groups. In panels **(D,E,G,H)**, error bars indicate ± S.D. (*n* = 3 biological replicates). In panel **(I)**, the data are the mean ± S.D. of six independent biological replicates. Student’s *t*-test, **P < 0.01.

According to the analysis of the promoter sequences (Supplementary Figure 7), it was predicted that *GhGPAT12*/*25* may be regulated by MYB or/and bHLH because of the existence of their binding sites. To further identify the potential upstream regulatory genes, MYB and bHLH transcription factors, which showed similar expression patterns to those of *GhGPAT12*/*25*, were screened based on the previous RNA-seq data of anther samples and other vegetative tissues (Zhang et al., 2015, 2020). Five pairs of homologous genes were found to be strictly expressed in anthers at the early developmental stages (Figure 10A). Subsequently, a yeast one-hybrid system (Y1H) was performed using two sequences that represent the most likely binding sites of MYB or/and bHLH TFs (Figure 10B). For the TFs, the study revealed that only *GhMYB80* (*GhMYB80-A*, *GH_A04G0015*) could directly bind to the W*AACC*A (MYB1AT) site in *GhGPAT12*/*25* promoters (Figure 10C). However, the other genes exhibited no or low binding activity on the SD/-Trp/-Leu/-His + 200mM 3-AT selective medium (Figure 10C).

GhMYB80 has been reported to share conserved sequences with that of *Arabidopsis* and *Brassica* and to rescue the male sterile phenotype of *atmyb80* in *Arabidopsis* (Xu et al., 2014). The qRT-PCR analysis was carried out to identify the expression pattern of *GhMYB80s* in various tissues; thus, the specific expression characteristic in anthers during the TTP stage was determined (Figure 10D). To explore their potential roles in cotton anther development and verify the regulatory relationship with *GhGPAT12*/*25*, a VIGS assay was used in TM-1 to transiently suppress the expression of *GhMYB80s*. A 300-bp fragment of the *GhMYB80-A* CDS from the non-conserved region was used in the (pCLCrVA)-based VIGS construct. As expected, pCLCrVA-*GhMYB80s* plants showed a significant decline in male fertility, characterized by 64%–93% of the anthers being shriveled (Figures 10E,F). The expressions of *GhMYB80s* in TTP stage anthers of the positive plants were down-regulated by approximately 59%–85% compared to the negative control plants (Figure 10G), implying that the corresponding phenotype was indeed due to the silence of *GhMYB80s*. Furthermore, the expression levels of *GhGPAT12*/*25* in *GhMYB80s*-slienced plants were found to be significantly reduced in positive anthers (Figure 10H). This suggested that *GhGPAT12*/*25* act downstream of *GhMYB80s* and might be activated by GhMYB80s.

The activation of the *GhGPAT12*/*25* promoters by the GhMYB80s protein was further confirmed via dual-luciferase (LUC) assays. In this system, the promoter sequence of *GhGPAT12* was constructed to the pGreenII0800-LUC reporter plasmid, and the CDS of *GhMYB80*-*A* (*GH_A04G0015*) was cloned into an effecter plasmid (pGreenII62-SK) and driven by a CaMV35S promoter. The *GhMYB80* transactivation level to the probable downstream promoter was measured by normalizing the signal of the luciferase reporter to that of Renilla luciferase (REN). The assay showed that the co-transformed effector-reporter exhibited a statistically significant higher-level transactivation in comparison with negative control (Figure 10I). This result indicated that *GhGPAT12* can be activated by GhMYB80-A. According to the high similarity of the *GhGPAT12*/*25* promoters (Supplementary Figure 7), it can be concluded that GhMYB80s are likely to directly bind to the specific elements in the *GhGPAT12*/*25* promoters and activate the expression of *GhGPAT12*/*25* for their subsequent functionalization.

## Discussion

### GhGPAT12/25 Are Required for the Anther Cuticle Formation and Pollen Exine Assembly

Heterosis utilization is an expectable goal in cotton genetic breeding. Male sterile lines are considered of great agricultural importance for the production of hybrids in order to improve the productivity and quality of cotton fiber (Zhu et al., 2008b). Few genes have been identified to be crucial for the formation of male organs in cotton (Wang and Li, 2009; Li et al., 2013, 2020; Liu et al., 2019a; Wang et al., 2019), and, therefore, the underlying regulatory mechanism controlling anther development and male sterility in cotton remains elusive.

In this research, GhGPAT12 and GhGPAT25, a paralogous gene pair, were identified encoding glycerol-3-phosphate acyltransferases in cotton (Figure 2). Evidences strongly suggesting a significant role of such pair in the anther cuticle formation and pollen development were also discovered (Figures 4–6). As genes specifically expressed in anthers, *GhGPAT12*/*25* are predominantly transcribed during early anther developmental stages (Figure 1). According to previous studies in *Arabidopsis* and rice, the expression characteristics of *GhGPAT12*/*25* are similar to many male sterility genes, represented by *MYB80* (Phan et al., 2011; Pan et al., 2020), *MS1*/*PTC1* (Wilson et al., 2001; Li et al., 2011), *CYP703A2*/*CYP703A3* (Morant et al., 2007; Yang et al., 2014), *CYP704B1*/*CYP704B2* (Dobritsa et al., 2009; Li et al., 2010), and *ACOS5*/*OsACOS12* (de Azevedo Souza et al., 2009; Li et al., 2016). These genes were reported to play essential roles in anther cuticle assembly and pollen exine development, indicating the potential functions of *GhGPAT12*/*25* in cotton anther development. The knockout of *GhGPAT12*/*25* mediated by CRISPR/Cas9 supported this hypothesis, thus yielding a completely male sterile mutant (Figure 3 and Supplementary Figures 2, 3).

The cytological observation and biochemical analysis of this research revealed that aberrant lipidic metabolism in *ghgpat12*/*25* anther affects the precise tapetum degeneration and the biosynthesis of essential precursors in anther cuticle and pollen exine (Figures 4–7, 9 and Supplementary Figure 6). The tapetal layer of *ghgpat12*/*25* could not undergo timely PCD, but displayed a series of unpredictable changes, such as lightly staining and swollen appearance, from the MC stage to the BNP stage (Supplementary Figures 6, 7). These abnormal morphologies are reminiscent of the phenotype of many male sterile lines, such as *atgpat1* (Zheng et al., 2003) and *lap5lap6* (Kim et al., 2010) in *Arabidopsis*; *dpw* (Shi et al., 2011), *dpw3* (Mondol et al., 2019), and *cyp704b2* (Li et al., 2010) in rice; and *zmms33* (Zhang et al., 2018; Zhu et al., 2019) in maize. Additionally, during the late developmental stage, the acute and abnormal degradative tapetum in *ghgpat12*/*25* anther and the aborted pollen grains leaving the atrophic anther locules (Figures 4J,L) indicate defects in synthesis and transport of lipidic precursors from the tapetum cell to the pollen wall surface in the mutant. These defects confirm the essential role of the cooperative action between tapetum cell and microspore for pollen development.

The anormogenesis of microspores is another characteristic in *ghgpat12*/*25*. Microspores in *ghgpat12*/*25* exhibited an irregular shape and abnormal exine (Figures 4–6). According to SEM detection, the pollen grains of *ghgpat12*/*25* were relatively smooth with no reticulate exine pattern and spines on the pollen surface (Figures 5G,H). These deficiencies were caused by the poor development of sexine (tectum and bacula) on the pollen wall (Figures 5G,H). Such development was exposed by the TEM analysis (Figures 6J,P,V). Altogether, these deficiencies in tapetal layer PCD and microsporogenesis lead to the male sterility, and these results may be predicted by the expression of *GhGPAT12*/*25* in both tapetal cells and pollen surface.

Despite the un-expression of *GhGPAT12*/*25* on anther epidermal cells shown by the RNA *in situ* hybridization experiment (Figure 1), *GhGPAT12*/*25* were also found to be critical for anther cuticle development (Figure 5). Anther cuticle is the protective barrier for the normal development of anthers and pollen grains in the anther locules (Piffanelli et al., 1998). As the downstream executor of anther lipid metabolism, GPAT is thought to be a committed enzyme for glycerol synthesis and an essential factor in the precise assembly of cuticle monomers (Li-Beisson et al., 2013). In the present study, SEM was performed to observe the anther surface of *ghgpat12*/*25*, thus discovering the seemingly lacking cuticle on anther surface (Figures 5E,F). This defect is consistent with the observations in *osgpat3* (Men et al., 2017) and *zmms33* (Zhang et al., 2018; Zhu et al., 2019) mutants. Moreover, this defect may be explained by the biochemical evidence establishing that the synthesis of wax monomers, such as alkanes and fatty acids, as well as several other metabolites (tocopherols, campesterols, and diterpenoids), was largely reduced in *ghgpat12*/*25* anthers (Figure 9).

Therefore, these series of concomitant defects in pollen exine and anther cuticle highlight that the sporopollenin and cuticle may share similar lipidic constituents supplied by tapetal layer cells, and *GhGPAT12*/*25* may act as important factors in affecting the normal functioning of tapetum during anther development.

### GhGPAT12/25 Have Conserved and Diversified Functions for Anther Development in Dicot

The sn-Glycerol-3-phosphate O-acyltransferase (GPAT, EC 2.3.1.15) is the first enzyme for the assembly of membrane and storage glycerolipids (Zheng et al., 2003). Previous studies revealed that GPATs can be distinguished by the positions (sn-1 or sn-2) that catalyze the transfer of an acyl group from acyl-CoA/acyl-ACP to glycerol-3-phosphate (G3P) and whether they have phosphorylation activity. These characteristics have been extensively illustrated in animals and plants (Yang et al., 2012). In general, different structural characteristics and catalytic performance determine the diverse functions of GPATs. The sn-1 acyltransferases were found to play important roles in the production of intracellular storage oil and to be represented by ATS1 (Nishida et al., 1993) and AtGPAT9 (Gidda et al., 2009) in *Arabidopsis*. The sn-2 acyltransferases are likely involved in the biosynthesis of extracellular lipid barrier polyesters, cutin and suberin, such as AtGPAT1–8 (Yang et al., 2012), BnGPAT4 (Chen et al., 2014), OsGPAT3 (Men et al., 2017), ZmMS33 (Zhang et al., 2018; Zhu et al., 2019), and SlGPAT6 (Petit et al., 2016). Despite the scarce information about the molecular structure and biosynthetic steps of lipid polymers, the synthesis of cutin glycerolipid is produced via a variant set of reactions that store lipid biosynthesis (Yang et al., 2010).

Based on the protein conserved domain analysis, GhGPAT12/25 were identified as glycerol-3-phosphate acyltransferases (Figure 2). Phylogenetic tree analysis implied that GhGPAT12/25 were assigned to clade I and considered to be sn-2 acyltransferases homologous to AtGPAT1, indicating the potential roles of GhGPAT12/25 in polyester synthesis (Figure 2B). Sequence alignment showed that GhGPAT12/25 and homologous members in other species share common conserved boxes (AT-I to AT-IV) in acyltransferase (AT) domain, with the corresponding catalytic and binding residues in all sequences (Figure 2C). In addition, the HAD-like domain located in the N-terminal region of GPATs is thought to be a typical reference for distinguishing the existence of phosphohydrolytic activity. Like AtGPAT1/2/3/5/7, GhGPAT12/25 may not have this activity due to the absence of pivotal sites necessary for substrates binding and catalysis in DXD and GDXXXD motif, and they were predicted to acylate the sn-2 position of G3P and produce lysophosphatidic acid (LPA) (Figure 2C). In clade III, AtGPAT4, AtGPAT6, and AtGPAT8 contained intact activity sites in these motifs (Figure 2C) and functioned as unique bifunctional enzymes with both acyltransferase and phosphatase activity, producing sn-2 MAC (monoacylglycerol) rather than LPA (Yang et al., 2012).

*OsGPAT3* and *ZmMS33* have been reported to be indispensable for male reproduction (Men et al., 2017; Zhang et al., 2018; Zhu et al., 2019), but the functions of their homologous genes in *Arabidopsis* (*AtGPAT2*/*3*) are not yet clear (Yang et al., 2012). This evidenced the functional differentiation of GPAT in dicotyledon and monocotyledon. Notably, AtGPAT1/6 are considered as crucial factors to anther and tapetum development in *Arabidopsis* (Zheng et al., 2003; Li et al., 2012). Similar to its ortholog AtGPAT1 in *Arabidopsis*, GhGPAT12/25 were also found to benefit male fertility in cotton, indicating their relatively conserved functions in dicotyledon. However, unlike *AtGPAT1*, which expresses in flower buds and siliques, causing a partial male sterility with defective pollen grains and normal anther cuticle with loss of function (Zheng et al., 2003), *GhGPAT12*/*25* are specifically expressed in the early developmental anthers (Figure 1) and their mutants are completely male sterile with abnormal tapetum, immature exine, and a lack of cuticle (Figures 3–7), suggesting the diversified functions of GPAT during evolution. Altogether, these results reflect the conserved and diversified function of GhGPAT12/25 in male reproductive development. However, it is worth noting that more research on GPAT is needed to explore their specific evolutionary mechanism in dicots and monocots.

### A Potential Regulatory Pathway of *GhGPAT12*/*25* in Male Sterility

As an evolutionarily conserved gene in various plants, *MYB80* is located downstream of *AMS* to control the anther development and cuticle formation. MYB80 is considered to directly or indirectly regulate the expression of a series of functional genes, which contribute to the synthesis or transport of lipidic components required for anther development, such as *CYP703A2*, *MS2*, *LAP5*, and *LAP6* (Phan et al., 2011; Xiong et al., 2016; Lu et al., 2020). In addition, further genetic and biochemistry analysis have revealed that MYB80 directly activates the expression of *MS1* and *CYP703A2* by binding to the core motifs (AACC) located on the promoters (Xiong et al., 2016; Lu et al., 2020).

In this study, GhGPAT12/25 were found to be key enzymes in the synthetic pathway of lipidic monomers required for the formation of anther cuticle and pollen exine (Figures 5, 6). Their upstream regulator was then predicted to be GhMYB80s because of the similar expression patterns of them and the MYB1AT motif (CT*AACC*A) located on the promoter of *GhGPAT12*/*25* (Figure 10B). Y1H, dual luciferase, and VIGS assays, which were performed to investigate the potential regulatory mechanism of *GhGPAT12*/*25* in anther development, demonstrated that GhMYB80s are likely to directly bind to the core motif of *GhGPAT12*/*25* promoter, thereby activating the expression of *GhGPAT12*/*25* (Figures 10C,E–I). These results preliminarily reveal the regulatory mechanism of *GhGPAT12*/*25* in the process of anther cuticle formation and highlight the evolutionary conservation role of MYB80 in cotton anther development.

Indeed, except for the effects of MYB80 on anther cuticle formation, many MYB TFs, such as MYB16, MYB30, MYB94, MYB96, etc., were found to be crucial manipulators for the regulation process of cuticle synthesis in vegetative organs by regulating the expression of downstream lipidic-related genes (Oshima and Mitsuda, 2013; Lee et al., 2016; Zhang et al., 2019). Moreover, these downstream genes are mainly *CER1*, *LACS2*, *KCS1*, *KCS2*, *KCS6*, *KCR1*, and *CER3*, which are also involved in the formation of cutin and wax in anthers, thus implying that these genes may be regulated by diverse MYBs in different synthesis pathways of cuticle due to their ubiquitous expression in various tissues (Seo et al., 2011; Lee et al., 2016). Therefore, as key enzymes in anther cuticle formation pathway, *GhGPAT12*/*25* may also be regulated by MYBs. Altogether, these discussions ratify the underlying regulatory mechanism of *GhGPAT12*/*25* discovered in this research.

In view of our transcriptome analysis showing the significantly differential down-regulation of a large number of lipid biosynthetic and glycerol transport genes (*SNH1*, *CER1*, *CER2*, *CER3*, *CER6*, *LACS1*, *LTPG1*, *LTPG6*, *ABCG10*, *ABCG14*, and *ABCG25*, etc.) in *ghgpat12*/*25* anthers (Figure 8B and Supplementary Table 1), it can be speculated that *GhGPAT12*/*25* likely function in modulating the synthesis and transport of lipidic framework during anther development, while their loss of function leads to the disturbances of these processes.

In previous researches, SNH1, CER1, CER2, CER3, and CER6 are considered to contribute to the production of epicuticular wax (Millar et al., 1999; Fiebig et al., 2000; Ariizumi et al., 2003; Broun et al., 2004; Haslam et al., 2012) and not affect the content and composition of cutin. LACS1 is an acyl-CoA synthetase that acts on long-chain and very-long-chain fatty acids, and it is important for the biosynthesis and assembly of both cuticular wax and cutin polyester in *Arabidopsis* (Lu et al., 2009). Therefore, according to the potential role of *GhGPAT12*/*25* for the formation of cutin monomers, we speculated that the down-regulation of these wax-related genes may be due to the lack of cutin matrix that supports the correct incorporation of intracuticular and epicuticular waxes into the cuticle backbone. In other words, the failed cutin synthesis process affected the production of wax components. This hypothesis was confirmed by the reduction of wax components in *ghgpat12*/*25* anthers (Figure 9).

Moreover, the lipidic monomers synthesized in tapetal cells need to be transported to the anther epidermis. This process requires the participation of ABCG transporters for the channeling of intracellular lipids through the plasma membrane and LTP(G)s responsible for exporting lipidic monomers to pass through the apoplastic compartment or hydrophilic cell wall (Edstam and Edqvist, 2014). Several *ABCGs* and *LTP(G)s* were found to be down-regulated in *ghgpat12*/*25* anthers (Figure 8B and Supplementary Table 1); among them, *LTPG1* has been reported to bind to lipids and to function as a component of the cuticular lipid export machinery in *Arabidopsis*. The mutation of *LTPG1* causes the alterative compositions of cuticular lipid on epidermis (Debono et al., 2009). Meanwhile, *LTPG6*, which is abundantly expressed in pollen, has previously been found to affect the pollen morphology, ovule fertility, and seed formation (Edstam and Edqvist, 2014). *LTPG26* is considered a young anther-specific gene, but its function is still unclear. Additionally, several genes, such as *DRN1* (Dhar et al., 2020), *LTP4* (Deeken et al., 2016), *ABCG14* (Wang et al., 2017), and *ABCG25* (Kuromori et al., 2010), were reported to be responsible for systemic resistance, especially for bacterial/fungus pathogens. It is worth considering that, although there is not enough evidence, these genes may exert resistance by transporting lipidic components used to synthesize cuticle. Other genes are mainly functionally ambiguous in *Arabidopsis* because of their unique effect characteristics. For example, ABCGs are half-transporters and require dimerization to function. Different dimer combinations may have diverse substrates, thus performing different functions (McFarlane et al., 2010). Therefore, their down-regulation in *ghgpat12*/*25* may imply their potential roles in anther cuticle development, and it is indispensable to explore the detailed biological functions of ABCGs and LTP(G)s members in future studies, especially their effects in anther development.

In conclusion, a pair of paralogs *GhGPAT12*/*25* specifically expressed in early developmental stage tapetum and microspores were identified in this research. CRISPR/Cas9-mediated knockout and gene regulatory mechanism analysis demonstrated that GhGPAT12/25 are indispensable enzymes acting downstream of GhMYB80s and contributing to the anther cuticle formation and pollen exine development by synthesizing glycerol framework. Moreover, the loss of function of GhGPAT12/25 affects the expression of genes related to the synthesis and transport of cutin and wax, thus leading to the male sterile phenotype in *ghgpat12*/*25*. This study discovered pivotal genes controlling male sterility and provided important insights into the regulatory mechanism underlying anther development in cotton.

## Data Availability Statement

The original contributions presented in the study are included in the article/Supplementary Material, further inquiries can be directed to the corresponding author/s.

## Author Contributions

SY and HLW conceived and designed the experiments. HLW, MZ, PH, AW, and JZ performed the experiments. HLW, MZ, QM, LM, HTW, JL, and XF analyzed the data. MZ wrote the manuscript. SY and HLW revised the manuscript. All authors reviewed and approved the final manuscript.

## Conflict of Interest

The authors declare that the research was conducted in the absence of any commercial or financial relationships that could be construed as a potential conflict of interest.

## Footnotes

1 www.miaolingbio.com

2 https://biodb.swu.edu.cn/qprimerdb

3 https://web.expasy.org/protparam/

4 http://pfam.xfam.org/

5 http://www.ncbi.nlm.nih.gov/

6 http://www.Arabidopsis.org/

7 http://www.megasotware.net/

8 http://crispr.hzau.edu.cn/CRISPR/

9 http://bioinformatics.psb.ugent.be/webtools/plantcare/html/

10 www.omicshare.com/tools

11 https://string-db.org/

## Supplementary Material

Supplementary Figure 1 Amino acid sequences of GhGPAT12/25.

Supplementary Figure 2 Variation information of T_0_ transgenic lines.

Supplementary Figure 3 Variation information of T_2_ transgenic lines.

Supplementary Figure 4 qRT-PCR analysis of anthers in HM-1 and *GhGPAT12*/*25.*

Supplementary Figure 5 Weight/surface area ratio for the anthers in WT and *ghgpat12*/*25.*

Supplementary Figure 6 Amounts of alcohols in WT and ghgpat12/25 anthers.

Supplementary Figure 7 Promoter sequences of *GhGPAT12*/*25.*

Supplementary Table 1 DEGs involved in the synthesis and transport of lipidic monomers.

Supplementary Table 2 Wax monomers in WT and *ghgpat12*/*25* anthers.

Supplementary Table 3 Primers used in this study.

## References

[B1] Aarts M. G. Keijzer C. J. Stiekema W. J. Pereira A. (1995). Molecular characterization of the CER1 gene of Arabidopsis involved in epicuticular wax biosynthesis and pollen fertility. *Plant Cell* 7 2115–2127. 10.1105/tpc.7.12.2115 8718622 PMC161066

[B2] Ariizumi T. Hatakeyama K. Hinata K. Sato S. Kato T. Tabata S. (2003). A novel male-sterile mutant of *Arabidopsis thaliana*, faceless pollen-1, produces pollen with a smooth surface and an acetolysis-sensitive exine. *Plant Mol. Biol.* 53 107–116. 10.1023/b:plan.0000009269.97773.70 14756310

[B3] Broun P. Poindexter P. Osborne E. Jiang C. Z. Riechmann J. L. (2004). WIN1, a transcriptional activator of epidermal wax accumulation in *Arabidopsis*. *Proc. Natl. Acad. Sci. U S A.* 101 4706–4711. 10.1073/pnas.0305574101 15070782 PMC384811

[B4] Chen W. Yu X. H. Zhang K. Shi J. De Oliveira S. Schreiber L. (2011). Male Sterile2 encodes a plastid-localized fatty acyl carrier protein reductase required for pollen exine development in *Arabidopsis*. *Plant Physiol.* 157 842–853. 10.1104/pp.111.181693 21813653 PMC3192575

[B5] Chen X. Chen G. Truksa M. Snyder C. L. Shah S. Weselake R. J. (2014). Glycerol-3-phosphate acyltransferase 4 is essential for the normal development of reproductive organs and the embryo in *Brassica napus*. *J. Exp. Bot.* 65 4201–4215. 10.1093/jxb/eru199 24821955 PMC4112632

[B6] Cui Y. Ma J. Liu G. Wang N. Pei W. Wu M. (2019). Genome-wide identification, sequence variation, and expression of the Glycerol-3-Phosphate Acyltransferase (GPAT) gene family in gossypium. *Front. Genet.* 10:116. 10.3389/fgene.2019.00116 PMC6391866 30842789

[B7] de Azevedo Souza C. Kim S. S. Koch S. Kienow L. Schneider K. McKim S. M. (2009). A novel fatty Acyl-CoA synthetase is required for pollen development and sporopollenin biosynthesis in *Arabidopsis*. *Plant Cell* 21 507–525. 10.1105/tpc.108.062513 19218397 PMC2660628

[B8] Debono A. Yeats T. H. Rose J. K. Bird D. Jetter R. Kunst L. (2009). *Arabidopsis* LTPG is a glycosylphosphatidylinositol-anchored lipid transfer protein required for export of lipids to the plant surface. *Plant Cell* 21 1230–1238. 10.1105/tpc.108.064451 19366900 PMC2685631

[B9] Deeken R. Saupe S. Klinkenberg J. Riedel M. Mueller T. D. (2016). The nsLTP AtLtp1-4 is involved in suberin formation of *Arabidopsis thaliana* crown galls. *Plant Physiol.* 172:1911. 10.1104/pp.16.01486 PMC5100791 27688623

[B10] Dhar N. Caruana J. Erdem I. Raina R. (2020). An *Arabidopsis* DISEASE RELATED NONSPECIFIC LIPID TRANSFER PROTEIN 1 is required for resistance against various phytopathogens and tolerance to salt stress. *Gene* 753:144802. 10.1016/j.gene.2020.144802 32454178

[B11] Dobritsa A. A. Shrestha J. Morant M. Pinot F. Matsuno M. Swanson R. (2009). CYP704B1 is a long-chain fatty acid omega-hydroxylase essential for sporopollenin synthesis in pollen of *Arabidopsis*. *Plant Physiol.* 151 574–589. 10.1104/pp.109.144469 19700560 PMC2754625

[B12] Edstam M. M. Edqvist J. (2014). Involvement of GPI-anchored lipid transfer proteins in the development of seed coats and pollen in *Arabidopsis thaliana*. *Physiol. Plant* 152 32–42. 10.1111/ppl.12156 24460633

[B13] Feng B. Lu D. Ma X. Peng Y. Sun Y. Ning G. (2012). Regulation of the *Arabidopsis* anther transcriptome by DYT1 for pollen development. *Plant J.* 72 612–624. 10.1111/j.1365-313X.2012.05104.x 22775442

[B14] Fiebig A. Mayfield J. A. Miley N. L. Chau S. Fischer R. L. Preuss D. (2000). Alterations in CER6, a gene identical to CUT1, differentially affect long-chain lipid content on the surface of pollen and stems. *Plant Cell* 12 2001–2008. 10.1105/tpc.12.10.2001 11041893 PMC149136

[B15] Gidda S. K. Shockey J. M. Rothstein S. J. Dyer J. M. Mullen R. T. (2009). *Arabidopsis thaliana* GPAT8 and GPAT9 are localized to the ER and possess distinct ER retrieval signals: functional divergence of the dilysine ER retrieval motif in plant cells. *Plant Physiol. Biochem.* 47 867–879. 10.1016/j.plaphy.2009.05.008 19539490

[B16] Goldberg R. B. Sanders P. M. Beals T. P. (1995). A novel cell-ablation strategy for studying plant development. *Philos. Trans. R. Soc. Lond. B Biol. Sci.* 350 5–17. 10.1098/rstb.1995.0131 8577850

[B17] Haslam T. M. Manas-Fernandez A. Zhao L. Kunst L. (2012). Arabidopsis ECERIFERUM2 is a component of the fatty acid elongation machinery required for fatty acid extension to exceptional lengths. *Plant Physiol.* 160 1164–1174. 10.1104/pp.112.201640 22930748 PMC3490600

[B18] Heredia A. (2003). Biophysical and biochemical characteristics of cutin, a plant barrier biopolymer. *Biochim. Biophys. Acta* 1620 1–7. 10.1016/s0304-4165(02)00510-x 12595066

[B19] Hu Y. Chen J. Fang L. Zhang Z. Ma W. Niu Y. (2019). Gossypium barbadense and *Gossypium hirsutum* genomes provide insights into the origin and evolution of allotetraploid cotton. *Nat. Genet.* 51 739–748. 10.1038/s41588-019-0371-375 30886425

[B20] Kim S. S. Grienenberger E. Lallemand B. Colpitts C. C. Kim S. Y. Souza Cde A. (2010). LAP6/POLYKETIDE SYNTHASE A and LAP5/POLYKETIDE SYNTHASE B encode hydroxyalkyl alpha-pyrone synthases required for pollen development and sporopollenin biosynthesis in *Arabidopsis thaliana*. *Plant Cell* 22 4045–4066. 10.1105/tpc.110.080028 21193570 PMC3027170

[B21] Kolattukudy P. E. (2001). Polyesters in higher plants. *Adv. Biochem. Eng. Biotechnol.* 71 1–49. 10.1007/3-540-40021-4_1 11217409

[B22] Kouchi H. Hata S. (1993). Isolation and characterization of novel nodulin cDNAs representing genes expressed at early stages of soybean nodule development. *Mol. Gen. Genet.* 238 106–119. 10.1007/BF00279537 7683079

[B23] Kunst L. Samuels A. L. (2003). Biosynthesis and secretion of plant cuticular wax. *Prog. Lipid Res.* 42 51–80. 10.1016/s0163-7827(02)00045-40 12467640

[B24] Kuromori T. Miyaji T. Yabuuchi H. Shimizu H. Sugimoto E. Kamiya A. (2010). ABC transporter AtABCG25 is involved in abscisic acid transport and responses. *Proc. Natl. Acad. Sci. U S A.* 107 2361–2366. 10.1073/pnas.0912516107 20133881 PMC2836683

[B25] Lee S. B. Kim H. U. Suh M. C. (2016). MYB94 and MYB96 additively activate cuticular wax biosynthesis in *Arabidopsis*. *Plant Cell Physiol.* 57 2300–2311. 10.1093/pcp/pcw147 27577115

[B26] Li F. S. Phyo P. Jacobowitz J. Hong M. Weng J. K. (2019a). The molecular structure of plant sporopollenin. *Nat. Plants* 5 41–46. 10.1038/s41477-018-0330-337 30559416

[B27] Li J. Wang M. Li Y. Zhang Q. Lindsey K. Daniell H. (2019b). Multi-omics analyses reveal epigenomics basis for cotton somatic embryogenesis through successive regeneration acclimation process. *Plant Biotechnol. J.* 17 435–450. 10.1111/pbi.12988 29999579 PMC6335067

[B28] Li H. Pinot F. Sauveplane V. Werck-Reichhart D. Diehl P. Schreiber L. (2010). Cytochrome P450 family member CYP704B2 catalyzes the {omega}-hydroxylation of fatty acids and is required for anther cutin biosynthesis and pollen exine formation in rice. *Plant Cell* 22 173–190. 10.1105/tpc.109.070326 20086189 PMC2828706

[B29] Li H. Yuan Z. Vizcay-Barrena G. Yang C. Liang W. Zong J. (2011). PERSISTENT TAPETAL CELL1 encodes a PHD-finger protein that is required for tapetal cell death and pollen development in rice. *Plant Physiol.* 156 615–630. 10.1104/pp.111.175760 21515697 PMC3177263

[B30] Li N. Zhang D. S. Liu H. S. Yin C. S. Li X. X. Liang W. Q. (2006). The rice tapetum degeneration retardation gene is required for tapetum degradation and anther development. *Plant Cell* 18 2999–3014. 10.1105/tpc.106.044107 17138695 PMC1693939

[B31] Li X. C. Zhu J. Yang J. Zhang G. R. Xing W. F. Zhang S. (2012). Glycerol-3-phosphate acyltransferase 6 (GPAT6) is important for tapetum development in *Arabidopsis* and plays multiple roles in plant fertility. *Mol. Plant* 5 131–142. 10.1093/mp/ssr057 21746699

[B32] Li Y. Beisson F. Koo A. J. Molina I. Pollard M. Ohlrogge J. (2007). Identification of acyltransferases required for cutin biosynthesis and production of cutin with suberin-like monomers. *Proc. Natl. Acad. Sci. U S A.* 104 18339–18344. 10.1073/pnas.0706984104 17991776 PMC2084344

[B33] Li Y. Jiang J. Du M. L. Li L. Wang X. L. Li X. B. (2013). A cotton gene encoding MYB-like transcription factor is specifically expressed in pollen and is involved in regulation of late anther/pollen development. *Plant Cell Physiol.* 54 893–906. 10.1093/pcp/pct038 23447105

[B34] Li Y. Li D. Guo Z. Shi Q. Xiong S. Zhang C. (2016). OsACOS12, an orthologue of *Arabidopsis* acyl-CoA synthetase5, plays an important role in pollen exine formation and anther development in rice. *BMC Plant Biol.* 16:256. 10.1186/s12870-016-0943-949 PMC5117612 27871243

[B35] Li Y. Li L. Wang Y. Wang Y. C. Wang N. N. Lu R. (2020). Pollen-specific protein PSP231 activates callose synthesis to govern male gametogenesis and pollen germination. *Plant Physiol.* 184 1024–1041. 10.1104/pp.20.00297 PMC7536655 32663166

[B36] Li-Beisson Y. Shorrosh B. Beisson F. Andersson M. X. Arondel V. Bates P. D. (2013). Acyl-lipid metabolism. *Arabidopsis Book* 11:e0161. 10.1199/tab.0161 PMC3563272 23505340

[B37] Liu F. Ma L. Wang Y. Li Y. Zhang X. Xue F. (2019a). GhFAD2-3 is required for anther development in Gossypium hirsutum. *BMC Plant Biol.* 19:393. 10.1186/s12870-019-2010-2019 PMC6734329 31500565

[B38] Liu Q. Wang C. Jiao X. Zhang H. Song L. Li Y. (2019b). Hi-TOM: a platform for high-throughput tracking of mutations induced by CRISPR/Cas systems. *Sci. China Life Sci.* 62 1–7. 10.1007/s11427-018-9402-9409 30446870

[B39] Lu J. Y. Xiong S. X. Yin W. Teng X. D. Lou Y. Zhu J. (2020). MS1, a direct target of MS188, regulates the expression of key sporophytic pollen coat protein genes in *Arabidopsis*. *J. Exp. Bot.* 71 4877–4889. 10.1093/jxb/eraa219 32374882 PMC7410184

[B40] Lu S. Song T. Kosma D. K. Parsons E. P. Rowland O. Jenks M. A. (2009). Arabidopsis CER8 encodes LONG-CHAIN ACYL-COA SYNTHETASE 1 (LACS1) that has overlapping functions with LACS2 in plant wax and cutin synthesis. *Plant J.* 59 553–564. 10.1111/j.1365-313X.2009.03892.x 19392700

[B41] McFarlane H. E. Shin J. J. Bird D. A. Samuels A. L. (2010). *Arabidopsis* ABCG transporters, which are required for export of diverse cuticular lipids, dimerize in different combinations. *Plant Cell* 22 3066–3075. 10.1105/tpc.110.077974 20870961 PMC2965547

[B42] Men X. Shi J. Liang W. Zhang Q. Lian G. Quan S. (2017). Glycerol-3-Phosphate Acyltransferase 3 (OsGPAT3) is required for anther development and male fertility in rice. *J. Exp. Bot.* 68 513–526. 10.1093/jxb/erw445 28082511 PMC6055571

[B43] Millar A. A. Clemens S. Zachgo S. Giblin E. M. Taylor D. C. Kunst L. (1999). CUT1, an *Arabidopsis* gene required for cuticular wax biosynthesis and pollen fertility, encodes a very-long-chain fatty acid condensing enzyme. *Plant Cell* 11 825–838. 10.1105/tpc.11.5.825 10330468 PMC144219

[B44] Mondol P. C. Xu D. Duan L. Shi J. Wang C. Chen X. (2019). Defective Pollen Wall 3 (DPW3), a novel alpha integrin-like protein, is required for pollen wall formation in rice. *New Phytol.* 225 807–822. 10.1111/nph.16161 31486533

[B45] Morant M. Jorgensen K. Schaller H. Pinot F. Moller B. L. Werck-Reichhart D. (2007). CYP703 is an ancient cytochrome P450 in land plants catalyzing in-chain hydroxylation of lauric acid to provide building blocks for sporopollenin synthesis in pollen. *Plant Cell* 19 1473–1487. 10.1105/tpc.106.045948 17496121 PMC1913723

[B46] Nishida I. Tasaka Y. Shiraishi H. Murata N. (1993). The gene and the RNA for the precursor to the plastid-located glycerol-3-phosphate acyltransferase of *Arabidopsis thaliana*. *Plant Mol. Biol.* 21 267–277. 10.1007/BF00019943 7678766

[B47] Oshima Y. Mitsuda N. (2013). The MIXTA-like transcription factor MYB16 is a major regulator of cuticle formation in vegetative organs. *Plant Signal Behav.* 8:e26826. 10.4161/psb.26826 PMC4091352 24169067

[B48] Pan X. Yan W. Chang Z. Xu Y. Luo M. Xu C. (2020). OsMYB80 regulates anther development and pollen fertility by targeting multiple biological pathways. *Plant Cell Physiol.* 61 988–1004. 10.1093/pcp/pcaa025 32142141 PMC7217667

[B49] Panikashvili D. Savaldi-Goldstein S. Mandel T. Yifhar T. Franke R. B. Hofer R. (2007). The *Arabidopsis* DESPERADO/AtWBC11 transporter is required for cutin and wax secretion. *Plant Physiol.* 145 1345–1360. 10.1104/pp.107.105676 17951461 PMC2151707

[B50] Petit J. Bres C. Mauxion J. P. Tai F. W. Martin L. B. Fich E. A. (2016). The Glycerol-3-Phosphate acyltransferase GPAT6 from tomato plays a central role in fruit cutin biosynthesis. *Plant Physiol.* 171 894–913. 10.1104/pp.16.00409 27208295 PMC4902622

[B51] Phan H. A. Iacuone S. Li S. F. Parish R. W. (2011). The MYB80 transcription factor is required for pollen development and the regulation of tapetal programmed cell death in *Arabidopsis thaliana*. *Plant Cell* 23 2209–2224. 10.1105/tpc.110.082651 21673079 PMC3160043

[B52] Piffanelli P. Ross J. H. E. Murphy D. J. (1998). Biogenesis and function of the lipidic structures of pollen grains. *Sexual Plant Reprod.* 11 65–80. 10.1007/s004970050122

[B53] Pighin J. A. Zheng H. Balakshin L. J. Goodman I. P. Western T. L. Jetter R. (2004). Plant cuticular lipid export requires an ABC transporter. *Science* 306 702–704. 10.1126/science.1102331 15499022

[B54] Porebski S. Bailey L. G. Baum B. R. (1997). Modification of a CTAB DNA extraction protocol for plants containing high polysaccharide and polyphenol components. *Plant Mol. Biol. Reporter* 15 8–15. 10.1007/bf02772108

[B55] Scott R. J. Spielman M. Dickinson H. G. (2004). Stamen structure and function. *Plant Cell* 16(Suppl.), S46–S60. 10.1105/tpc.017012 15131249 PMC2643399

[B56] Seo P. J. Lee S. B. Suh M. C. Park M. J. Go Y. S. Park C. M. (2011). The MYB96 transcription factor regulates cuticular wax biosynthesis under drought conditions in *Arabidopsis*. *Plant Cell* 23 1138–1152. 10.1105/tpc.111.083485 21398568 PMC3082259

[B57] Shi J. Tan H. Yu X. H. Liu Y. Liang W. Ranathunge K. (2011). Defective pollen wall is required for anther and microspore development in rice and encodes a fatty acyl carrier protein reductase. *Plant Cell* 23 2225–2246. 10.1105/tpc.111.087528 21705642 PMC3160036

[B58] Sorensen A. M. Krober S. Unte U. S. Huijser P. Dekker K. Saedler H. (2003). The *Arabidopsis* ABORTED MICROSPORES (AMS) gene encodes a MYC class transcription factor. *Plant J.* 33 413–423. 10.1046/j.1365-313x.2003.01644.x 12535353

[B59] Uzair M. Xu D. Schreiber L. Shi J. Liang W. Jung K. H. (2020). PERSISTENT TAPETAL CELL2 is required for normal tapetal programmed cell death and pollen wall patterning. *Plant Physiol.* 182 962–976. 10.1104/pp.19.00688 31772077 PMC6997677

[B60] Wang S. Wang S. Sun Q. Yang L. Zhu Y. Yuan Y. (2017). A role of cytokinin transporter in arabidopsis immunity. *Mol. Plant Microbe Interact.* 30 325–333. 10.1094/MPMI-01-17-0011-R 28398838

[B61] Wang X. L. Li X. B. (2009). The GhACS1 gene encodes an acyl-CoA synthetase which is essential for normal microsporogenesis in early anther development of cotton. *Plant J.* 57 473–486. 10.1111/j.1365-313X.2008.03700.x 18826432

[B62] Wang Y. Li Y. He S. P. Gao Y. Wang N. N. Lu R. (2019). A cotton (Gossypium hirsutum) WRKY transcription factor (GhWRKY22) participates in regulating anther/pollen development. *Plant Physiol. Biochem.* 141 231–239. 10.1016/j.plaphy.2019.06.005 31195253

[B63] Wilson Z. A. Morroll S. M. Dawson J. Swarup R. Tighe P. J. (2001). The *Arabidopsis* MALE STERILITY1 (MS1) gene is a transcriptional regulator of male gametogenesis, with homology to the PHD-finger family of transcription factors. *Plant J.* 28 27–39. 10.1046/j.1365-313X.2001.01125.x 11696184

[B64] Xiong S. X. Lu J. Y. Lou Y. Teng X. D. Gu J. N. Zhang C. (2016). The transcription factors MS188 and AMS form a complex to activate the expression of CYP703A2 for sporopollenin biosynthesis in *Arabidopsis thaliana*. *Plant J.* 88 936–946. 10.1111/tpj.13284 27460657

[B65] Xu Y. Iacuone S. Li S. F. Parish R. W. (2014). MYB80 homologues in *Arabidopsis*, cotton and *Brassica*: regulation and functional conservation in tapetal and pollen development. *BMC Plant Biol.* 14:278. 10.1186/s12870-014-0278-273 PMC4205283 25311582

[B66] Yang W. Pollard M. Li-Beisson Y. Beisson F. Feig M. Ohlrogge J. (2010). A distinct type of glycerol-3-phosphate acyltransferase with sn-2 preference and phosphatase activity producing 2-monoacylglycerol. *Proc. Natl. Acad. Sci. U S A.* 107 12040–12045. 10.1073/pnas.0914149107 20551224 PMC2900678

[B67] Yang W. Simpson J. P. Li-Beisson Y. Beisson F. Pollard M. Ohlrogge J. B. (2012). A land-plant-specific glycerol-3-phosphate acyltransferase family in *Arabidopsis*: substrate specificity, sn-2 preference, and evolution. *Plant Physiol.* 160 638–652. 10.1104/pp.112.201996 22864585 PMC3461545

[B68] Yang X. J. Wu D. Shi J. X. He Y. Pinot F. Grausem B. (2014). Rice CYP703A3, a cytochrome P450 hydroxylase, is essential for development of anther cuticle and pollen exine. *J. Int. Plant Biol.* 56 979–994. 10.1111/jipb.12212 24798002

[B69] Zhang L. Luo H. Zhao Y. Chen X. Huang Y. Yan S. (2018). Maize male sterile 33 encodes a putative glycerol-3-phosphate acyltransferase that mediates anther cuticle formation and microspore development. *BMC Plant Biol.* 18:318. 10.1186/s12870-018-1543-1547 PMC6276174 30509161

[B70] Zhang M. Liu J. Ma Q. Qin Y. Wang H. Chen P. (2020). Deficiencies in the formation and regulation of anther cuticle and tryphine contribute to male sterility in cotton PGMS line. *BMC Genom.* 21:825. 10.1186/s12864-020-07250-7251 PMC7685665 33228563

[B71] Zhang T. Hu Y. Jiang W. Fang L. Guan X. Chen J. (2015). Sequencing of allotetraploid cotton (*Gossypium hirsutum* L. acc. TM-1) provides a resource for fiber improvement. *Nat. Biotechnol.* 33 531–537. 10.1038/nbt.3207 25893781

[B72] Zhang W. Sun Y. Timofejeva L. Chen C. Grossniklaus U. Ma H. (2006). Regulation of *Arabidopsis tapetum* development and function by DYSFUNCTIONAL TAPETUM1 (DYT1) encoding a putative bHLH transcription factor. *Development* 133 3085–3095. 10.1242/dev.02463 16831835

[B73] Zhang Y. L. Zhang C. L. Wang G. L. Wang Y. X. Qi C. H. Zhao Q. (2019). The R2R3 MYB transcription factor MdMYB30 modulates plant resistance against pathogens by regulating cuticular wax biosynthesis. *BMC Plant Biol.* 19:362. 10.1186/s12870-019-1918-1914 PMC6700842 31426743

[B74] Zheng Z. Xia Q. Dauk M. Shen W. Selvaraj G. Zou J. (2003). Arabidopsis AtGPAT1, a member of the membrane-bound glycerol-3-phosphate acyltransferase gene family, is essential for tapetum differentiation and male fertility. *Plant Cell* 15 1872–1887. 10.1105/tpc.012427 12897259 PMC167176

[B75] Zheng Z. Zou J. (2001). The initial step of the glycerolipid pathway: identification of glycerol 3-phosphate/dihydroxyacetone phosphate dual substrate acyltransferases in *Saccharomyces cerevisiae*. *J. Biol. Chem.* 276 41710–41716. 10.1074/jbc.M104749200 11544256

[B76] Zhu J. Chen H. Li H. Gao J. F. Jiang H. Wang C. (2008a). Defective in tapetal development and function 1 is essential for anther development and tapetal function for microspore maturation in *Arabidopsis*. *Plant J.* 55 266–277. 10.1111/j.1365-313X.2008.03500.x 18397379

[B77] Zhu W. Liu K. Wang X. D. (2008b). Heterosis in yield, fiber quality, and photosynthesis of okra leaf oriented hybrid cotton (*Gossypium hirsutum* L.). *Euphytica* 164:283. 10.1007/s10681-008-9732-9733

[B78] Zhu T. Wu S. Zhang D. Li Z. Xie K. An X. (2019). Genome-wide analysis of maize GPAT gene family and cytological characterization and breeding application of ZmMs33/ZmGPAT6 gene. *Theor Appl. Genet.* 132 2137–2154. 10.1007/s00122-019-03343-y 31016347

