# Supplementary material for: GhGPAT12/25 Are Essential for the Formation of Anther Cuticle and Pollen Exine in Cotton (Gossypium hirsutum L.)
Source: Front Plant Sci. 2021 May 13;12:667739. doi: 10.3389/fpls.2021.667739 (PMC8155372; doi:10.3389/fpls.2021.667739)
Supplement: Supplementary Figure 1 — Amino acid sequences of GhGPAT12/25. [file Data_Sheet_1.pdf]

Supplementary Material

1. Supplementary Figures

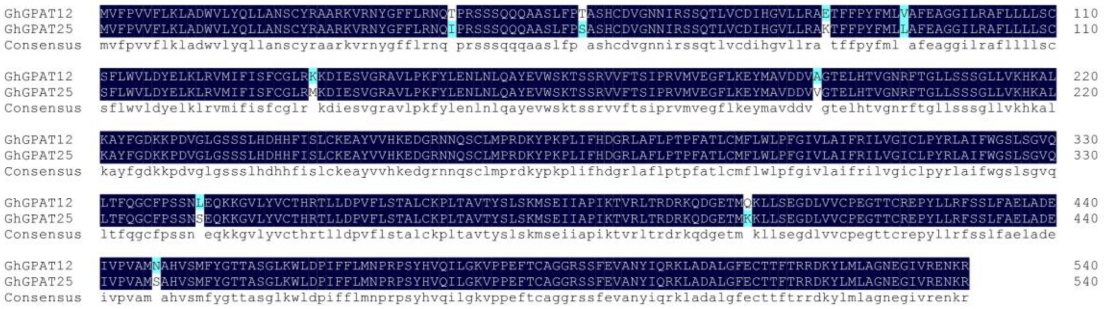

Figure S1. Amino acid sequences of GhGPAT12/25

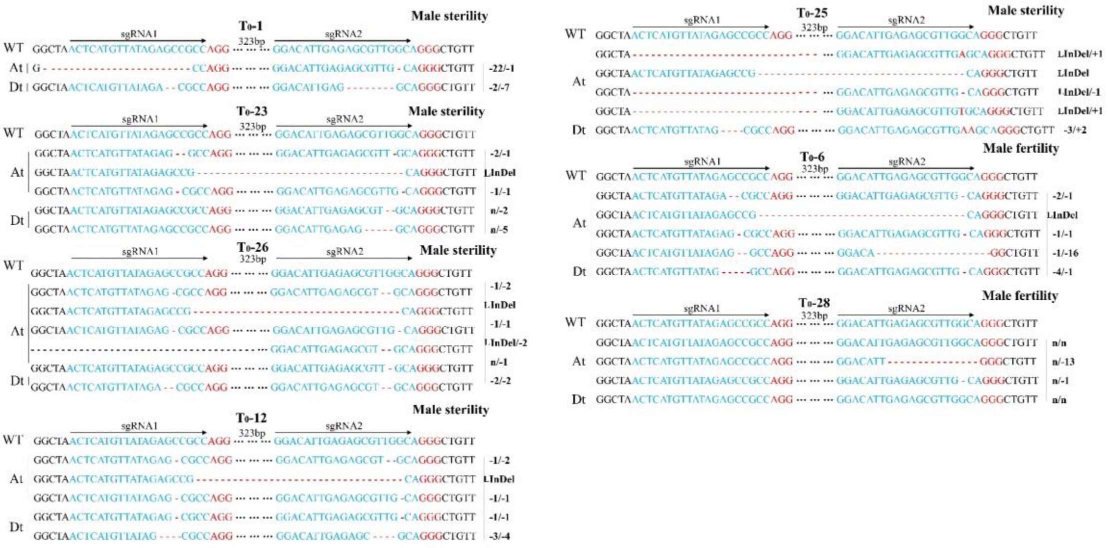

Figure S2. Variation information of T_0 transgenic lines

Variation characteristics of *GhGPAT12/25* in T_0 transgenic lines. The PAM sequence is shown in red. Deletions are denoted with red dashes. Insertions are shown as red letters. The mutation types are shown on the right.

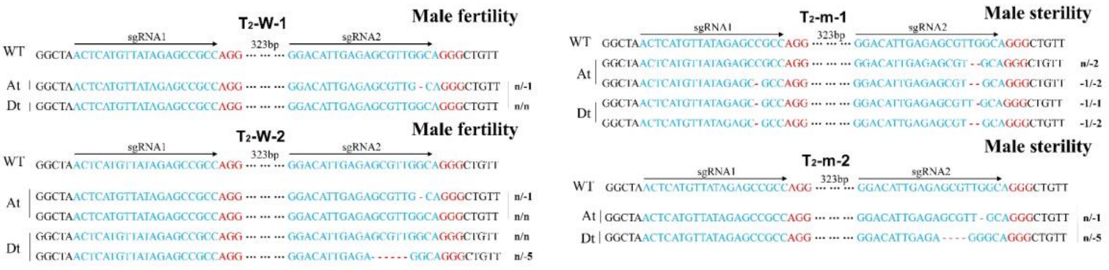

Figure S3. Variation information of T_2 transgenic lines

Variation characteristics of *GhGPAT12/25* in T_2 transgenic lines. The PAM sequence is shown in red. Deletions are denoted with red dashes. Insertions are shown as red letters. The mutation types are shown on the right.

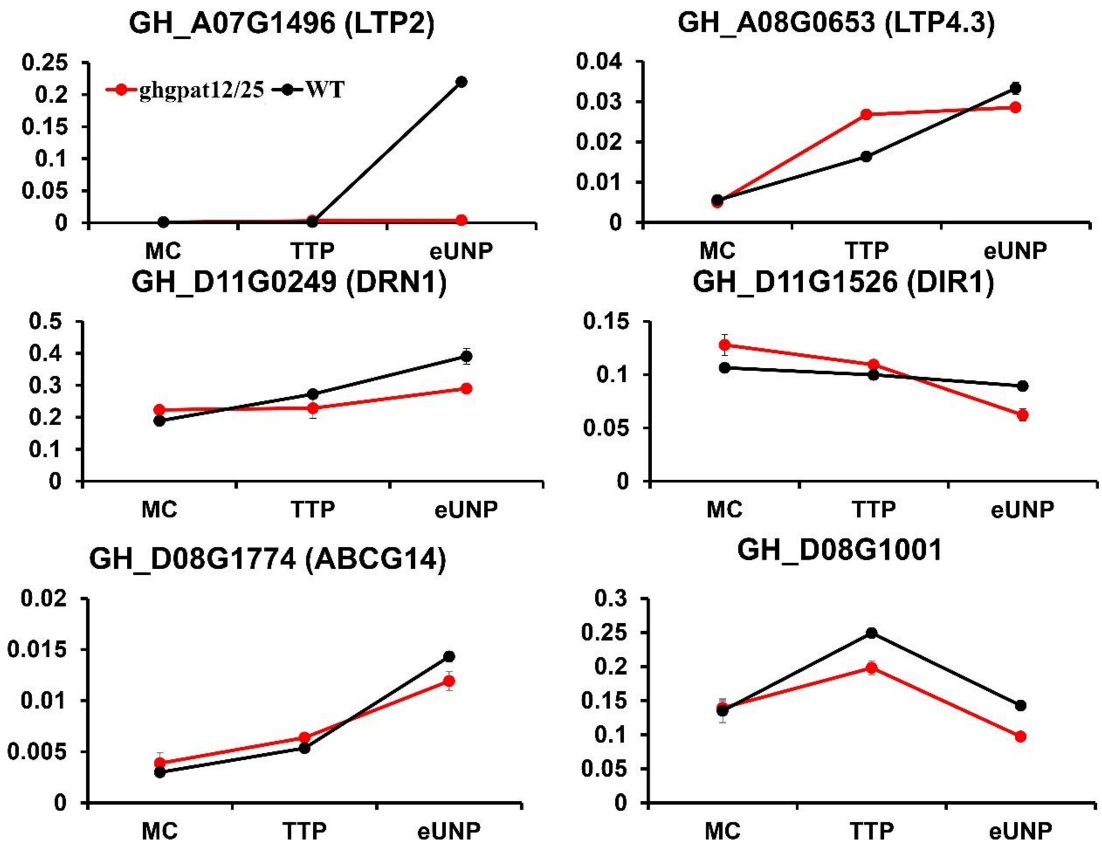

Figure S4. qRT-PCR analysis of anthers in HM-1 and *GhGPAT12/25*
Error bars indicate ± S.D. (n = 3 biological replicates).

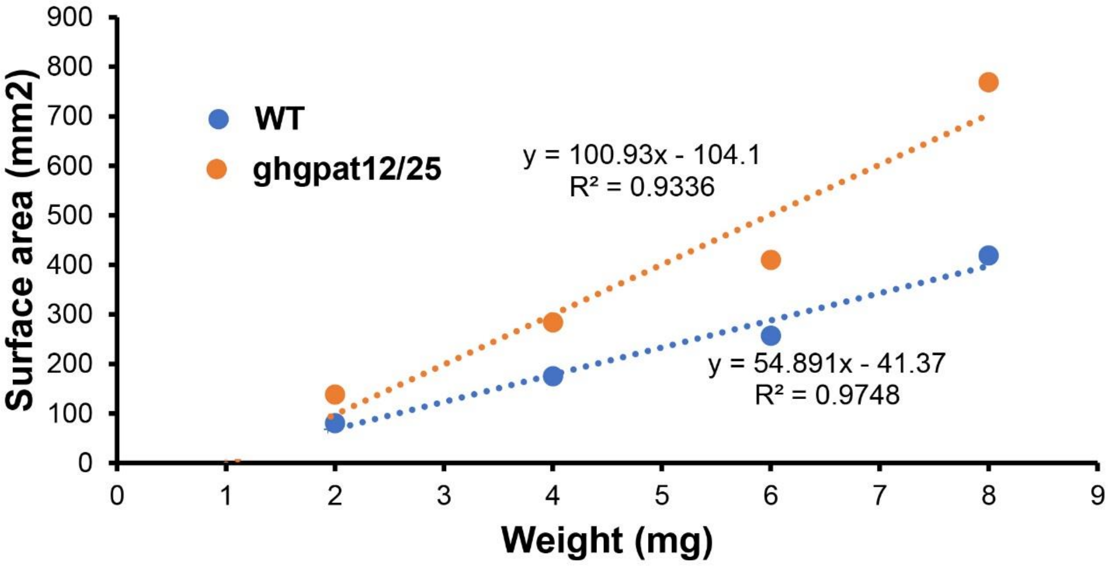

Figure S5. Weight/surface area ratio for the anthers in WT and *ghgpat12/25*

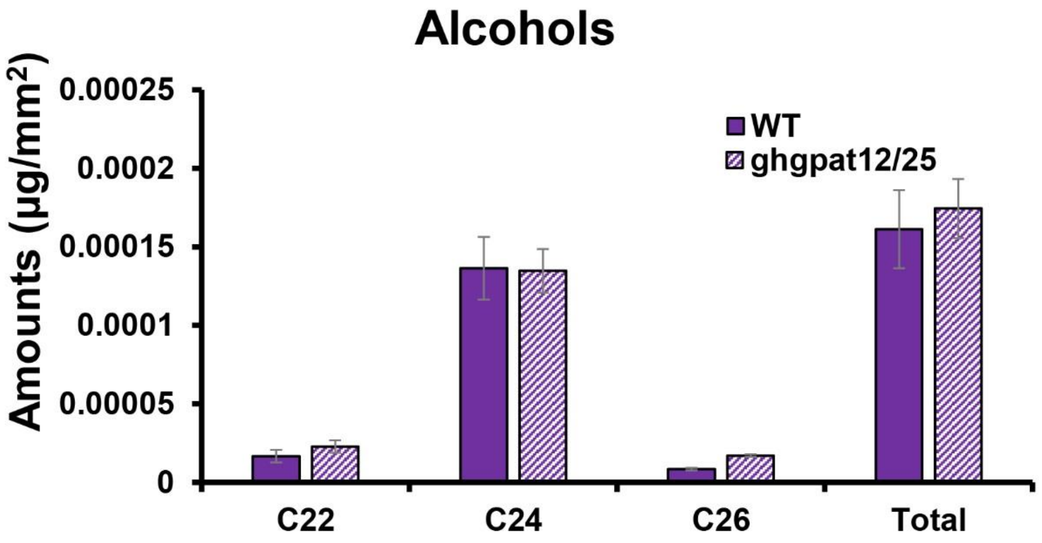

Figure S6. Amounts of alcohols in WT and ghgpat12/25 anthers

Error bars indicate ± S.D. (n = 3 biological replicates).

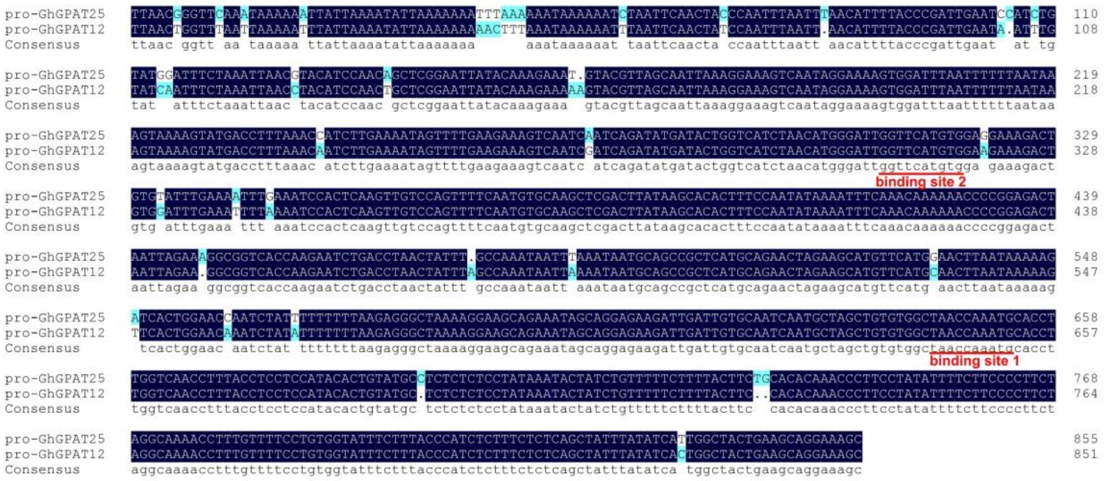

Figure S7. Promoter sequences of *GhGPAT12/25*

2. Supplementary Tables

Table S1. DEGs involved in the synthesis and transport of lipidic monomers

Gene_ID	Gene name	TTP_G	eUNP_G	TTP_W	eUNP_W
GH_A04G1689	LTPG1	52.25163	35.05408	49.30692	128.1104
GH_D02G0373	LTPG26	1.519867	1.442133	1.607767	6.485929
GH_D04G2037	LTPG1	41.77382	28.1363	31.08555	103.8551
GH_D05G0009	LTPG6	30.92339	11.08027	44.39338	28.41636
GH_D08G0861	LTPG6	2.1029	2.102405	3.624262	4.946266
GH_A07G1496	LTP2	1.17026	0.944894	1.441915	2.704203
GH_A08G0653	LTP4.3	10.30678	8.280373	9.662883	20.72389
GH_D11G0249	DRN1	2.92602	3.471072	2.599764	7.272293
GH_D11G1526	DIR1	47.60661	23.09848	40.3264	47.48631
GH_A07G0829	LTP4	1.384939	0.69124	2.932634	0.967108
GH_D05G2788	ABCG25	0.372885	4.198925	0.785245	32.38952
GH_D08G1774	ABCG14	1.422403	1.115311	0.855127	2.938392
GH_A09G0022	ABCG10	1.600212	0.760373	1.941431	1.7793
GH_D08G1001	TPRP-F1	25.60557	11.33095	18.58888	25.77962
GH_A06G1828	CER1	32.97263	34.63719	33.07731	69.83438
GH_D06G1860	CER1	61.37043	59.70032	57.02636	141.4015
GH_A13G2356	CER3	3.394307	1.440062	2.918918	3.436089
GH_A01G2012	KCS6	15.37379	8.4904	11.30078	19.26585
GH_D01G2106	KCS6	18.10364	11.40947	13.08407	29.54921
GH_A01G1242	CER2	1.169842	2.90913	0.719963	6.582086
GH_A07G1407	LACS1	54.01111	27.79236	101.8943	72.16169
GH_A10G0747	SHN1	10.44101	11.55987	5.378425	29.86295

Table S2. Wax monomers in WT and *ghgpat12/25* anthers

Wax monomers	WT	*ghgpat12/25*	Reduction
	Mean (μg/mm^2)	Mean (μg/mm^2)	(%)
**Alkanes**			
C24	0.002450253	0.001273369	48.03%
C25	0.018675468	0.001022894	94.52%
C26	0.001326034	0.00059741	54.95%
C27	0.016680704	0.005635182	66.22%
C28	0.000564537	0.000538108	4.68%
C29	0.006675801	0.001668531	75.01%
C30	0.000319596	8.96027E-05	71.96%
C31	0.006447804	0.000129136	98.00%
C32	0.000186132	4.77574E-05	74.34%
**Alcohols**			
C22	1.66062E-05	2.27857E-05	-37.21%
C24	0.000136337	0.000134656	1.23%
C26	8.38346E-06	1.69442E-05	-102.11%
**Fatty acids**			
C20	1.70885E-05	5.47809E-06	67.94%
C22	8.34975E-06	1.29278E-05	-54.83%
C24	0.000153514	1.28781E-05	91.61%
C26	5.84301E-05	6.63123E-06	88.65%
**Secondary metatolites**			
Tocopherols	0.000500093	0.000109768	78.05%
Campesterols	0.000296953	2.52698E-05	91.49%
Sitosterol	0.001306433	0.002404012	-84.01%
Diterpenoids	0.001874042	0.000302286	83.87%
Triterpenoids	0.00018816	0.000326259	-73.39%

Table S3. Primers used in this study

Name	Use	Primer (5′ →3′)
qGH_A06G1828-F	qRT-PCR	CATGGGGCATTGCAACTTCGAG
qGH_A06G1828-R	qRT-PCR	GAGGCAAATCCAAGGCGGAGAT
qGH_D06G1860-F	qRT-PCR	GCACCTAACCACACCTGAGTCG
qGH_D06G1860-R	qRT-PCR	GCCAGAGCAACCACAAGTACCA
qGH_A13G2356-F	qRT-PCR	GTCGACGCCATTTTGCATGAGG
qGH_A13G2356-R	qRT-PCR	ACGGCCGCGAAGATTGTAGAAA
qGH_A01G2012-F	qRT-PCR	CTGAACTTGAGCAACAACCCTA
qGH_A01G2012-R	qRT-PCR	AGCAGGAGGTAAACAAGTCTCT
qGH_D01G2106-F	qRT-PCR	TCCAACCATGGAGGCTGCTAGA
qGH_D01G2106-R	qRT-PCR	TGGCGTAGGAGAAAACAGACTGC
qGH_A07G1407-F	qRT-PCR	CCCACAGTTTGGTTTGTGTGCC
qGH_A07G1407-R	qRT-PCR	AATTGCTTTCAGCCGTTGAGCC
qGH_A01G1242-F	qRT-PCR	TGTTTCGAGGGTGGAAATAGCGG
qGH_A01G1242-R	qRT-PCR	CGCACACACTGGTTTCAATCCC
qGH_A10G0747-F	qRT-PCR	GCAGCCATTTTGATGAGCGGAC
qGH_A10G0747-R	qRT-PCR	TCCCTGCTCTCTTTTGCCACAC
qGH_A04G1689-F	qRT-PCR	CGCCGTCGAAGGAGTGTTGTAA
qGH_A04G1689-R	qRT-PCR	AGCCGACGGTAACTGAAACAGC
qGH_D02G0373-F	qRT-PCR	CTGCTGATGGGCCTGCTGTAAT
qGH_D02G0373-R	qRT-PCR	TGTTGATGAGTTGCTGCCTGGG
qGH_D04G2037-F	qRT-PCR	TTGGCCTTGATGGTGGTTTCCG
qGH_D04G2037-R	qRT-PCR	CCCGCCACTGAATTACAGCACT
qGH_D05G0009-F	qRT-PCR	GGTGTGTGTCTATGCCCTGGAA
qGH_D05G0009-R	qRT-PCR	TGCTATTATTATTTCTGCTGGG
qGH_D08G0861-F	qRT-PCR	TACGTTGGTGGACAGGCCAAAG
qGH_D08G0861-R	qRT-PCR	TGATGTTGAGGCCAACACTGGG
qGH_A07G1496-F	qRT-PCR	ATGACTCGCTCGTTCAACTTTC
qGH_A07G1496-R	qRT-PCR	AGCTGAGGCATGGGAGTAATTC
qGH_A08G0653-F	qRT-PCR	CCGAAAAAGTGCCACAACCACC
qGH_A08G0653-R	qRT-PCR	AGAGAGTAGGAAGCGATGCGGA
qGH_D11G0249-F	qRT-PCR	ATCCGCTGCTCTTCTTCTTACC
qGH_D11G0249-R	qRT-PCR	TGGGTAGGCTTGTGGCTCTTTG
qGH_D11G1526-F	qRT-PCR	TGGGTACAGTTACAGTGAAGTTGG
qGH_D11G1526-R	qRT-PCR	GCTTCCAGACCCTCATCGTTCA
qGH_A07G0829-F	qRT-PCR	TGTTTGGCGGTGGCTAACATCA
qGH_A07G0829-R	qRT-PCR	CTGCACCAGGACCAGCTTTCTT
qGH_D05G2788-F	qRT-PCR	GGCAGGAACAGCAACAGAGTCA
qGH_D05G2788-R	qRT-PCR	TGAGGAAACGCGAATACCGCAT
qGH_D08G1774-F	qRT-PCR	TGGAGCAAGTTGAGAGTACGGA
qGH_D08G1774-R	qRT-PCR	AACTGATACCACCGGCTTGTGC
qGH_A09G0022-F	qRT-PCR	GGCTACTAAACAAGGCAAAACA

qGH_A09G0022-R	qRT-PCR	ACAAAATTCGATCAAACAGCTC
qGH_D08G1001-F	qRT-PCR	GAGGGTTGGTTCACATTGGCCT
qGH_D08G1001-R	qRT-PCR	GGGGGTGTTTTCCCACAAGTGA
qGhMYB80-F	qRT-PCR	AGAAGAAGCTTTCAGGCACGGG
qGhMYB80-R	qRT-PCR	CACCTGTGGTGGTGCCAATGTA
qGhGPAT12-F	qRT-PCR	AAATCCTTGGAAAGGTGCCCCC
qGhGPAT12-R	qRT-PCR	ACTCAAATCCCAGTGCATCGGC
qGhGPAT25-F	qRT-PCR	GCAGGGCTGTTTTGCCAAAGTT
qGhGPAT25-R	qRT-PCR	GTGCAATTCAGTGCCTGCAACA
cGhGPAT12/25-F	Sequencing	ATGGTTTTTCCTGTGGTAT
cGhGPAT12/25-R	Sequencing	TCAATTTCTCTTATTTTCACGGA
pYES_GPAT12/25-F	Heterologous expression	ttggtaccgagctcggatccAACACAATGGTTTTTCCTGTGGTATTTTTG
pYES_GPAT12/25-R	Heterologous expression	cgagcggccgcTCAatgatgatgatgatgatgATTTCTCTTATTTTCACGGAC
GPAT12/25-T1as	CRISPR	GGCGGCTCTATAACATGAGTtgcaccagccgggaat
GPAT12/25-T1s	CRISPR	ACTCATGTTATAGAGCCGCCgttttagagctagaaata
GPAT12/25-T2as	CRISPR	TGCCAACGCTCTCAATGTCCtgcaccagccgggaat
GPAT12/25-inf-T2as	CRISPR	ttctagctctaaaacTGCCAACGCTCTCAATGTCC
Hi-GPAT12/25-F	Hi-TOM	CCTGTGGTATTTTTGAAGCTAGC
Hi-GPAT12/25-R	Hi-TOM	GGTTCTCAAGATAAAACTTTGGC
GhDYT1-F	Y1H	ggaggccagtgaattcATGGAATTCCTAACCGAGCT
GhDYT1-R	Y1H	cgagctcgatggatccTTAACTTACATGTACACAGG
GhAMS-F	Y1H	ggaggccagtgaattcATGATGATGCAGATAACCAT
GhAMS-R	Y1H	cgagctcgatggatccTCATGCCGCTTGCTTTGGTG
GhMYB80-F	Y1H	ggaggccagtgaattcATGGGTCGGATTCCATGTTG
GhMYB80-R	Y1H	cgagctcgatggatccTTATTCTTTGTTGTTAAAGG
GhTDF1-F	Y1H	ggaggccagtgaattcATGGTCAGACCTCCTTGTTG
GhTDF1-R	Y1H	cgagctcgatggatccCTATATGGTGGACCTTCTAA

GhbHLH91-F	Y1H	ggaggccagtgaattcATGAAGGTGTACGAGGAGAG
GhbHLH91-R	Y1H	cgagctcgatggatccCTAGAGAGAAGTACTACTAGTTGG
VA-F	VIGS	ATTTTGCGCCTGACTAGCCT
VA-R	VIGS	CGAATTTTCAACGTTGCATACA
VB-F	VIGS	ATGTACAGTTTAAAGAGTAGACG
VB-R	VIGS	ATTATCCAATATAATCAAGGTCATAC
vigs-GhMYB80-F	VIGS	tggcatgcctgcagactagtGGACACCCGAGGAAGACAAC
vigs-GhMYB80-R	VIGS	actagacctaggggcgcgccTTAGCAGGTGGAGCATTTCA
SK-GhMYB80-F	LUC	tagaactagtggatccATGGGTCGGATTCCATGTTG
SK-GhMYB80-R	LUC	cgggccccccctcgagTTATTCTTTGTTGTTAAAGG
LUC-GPAT12-F	LUC	cgggccccccctcgagAAATTTACATTCAAAATAAGTACA
LUC-GPAT12-R	LUC	tagaactagtggatccGGAAGGGTTTGTGTGCAG
GhGPAT12/25 antisense probe	RNA in situ hybridization	5’-DIG-UGCUCAAGCUGUAGGUCACGGCTGTCAAA-DIG-3’
GhGPAT12/25 sense probe	RNA in situ hybridization	5’-DIG-UUUGACAGCCGUGACCUACAGCUUGAGCA-DIG-3’
